# The International/Canadian hereditary angioedema guideline

**DOI:** 10.1186/s13223-025-00999-8

**Published:** 2026-01-28

**Authors:** Stephen Betschel, Karen Binkley, Rozita Borici-Mazi, Hugo Chapdelaine, Jacques Hébert, Amin Kanani, Paul K. Keith, Gina Lacuesta, Susan Waserman, William Yang, Valerie Palda, Kelly Lang-Robertson, Emel Aygören-Pürsün, Aleena Banerji, Jonathan A. Bernstein, Teresa Caballero, Henriette Farkas, Anete S. Grumach, Michihiro Hide, Connie Katelaris, Hilary J. Longhurst, Markus Magerl, Marc Riedl, Andrea Zanichelli, Bruce Zuraw, Adil Adatia, Jean-Nicolas Boursiquot, Lori Connors, Victoria Cook, Michelle Cooper, Lisa Fu, Parwinder Gill, Dawn Goodyear, Amanda Jagdis, Samira Jeimy, Chrystyna Kalicinsky, Palinder Kamra, Manstein Kan, Harold Kim, Benoît Laramée, Erika Lee, Eric Leith, Louis Marois, Christine McCusker, Andrew O’Keefe, Ibraheem Othman, Man-Chiu Poon, Charles St-Pierre, Peter Stepaniuk, Ellie Tsai, Kelsey Uminski

**Affiliations:** 1 University of Toronto, Toronto, ON Canada; 2 Oxford Allergy Clinic, London, ON Canada; 3 Université de Montréal, Montreal, QC Canada; 4 Cliniques Lacroix, Quebec City, QC Canada; 5 University of British Columbia, Vancouver, BC Canada; 6 McMaster University, Hamilton, ON Canada; 7 Dalhousie University, Halifax, NS Canada; 8 Ottawa Allergy Research Corporation, Ottawa, ON Canada; 9 Centre for Effective Practice, Toronto, ON Canada; 10 Goethe University Frankfurt, Frankfurt, Germany; 11 Massachusetts General Hospital, Boston, MA USA; 12 University of Cincinnati, Cincinnati, OH USA; 13 Hospital La Paz Institute for Health Research, Madrid, Spain; 14 Semmelweis University, Budapest, Hungary; 15 Centro Universitario Faculdade de Medicina ABC, Sao Paulo, Brazil; 16 Hiroshima City Hiroshima Citizens Hospital, Hiroshima City, Japan; 17 Campbelltown Hospital, Campbelltown, NSW Australia; 18 Western Sydney University, Richmond, NSW Australia; 19 Auckland City Hospital, Auckland, New Zealand; 20 University of Auckland, Auckland, New Zealand; 21 Institute of Allergology Charité, Universitätsmedizin Berlin, Berlin, Germany; 22 University of California - San Diego, San Diego, CA USA; 23 University of Milan, Milan, Italy; 24 University of Alberta, Edmonton, AB Canada; 25 Université Laval, Quebec City, QC Canada; 26 HAE Canada, Ottawa, ON Canada; 27 Dawson Road Family Medical Clinic, Guelph, ON Canada; 28 University of Calgary, Calgary, AB Canada; 29 Western University, London, ON Canada; 30 University of Manitoba, Winnipeg, MB Canada; 31 Memorial University, St John’s, NL Canada; 32 La Polyclinique Pierre-Le Gardeur, Terrebonne, QC Canada; 33 McGill University, Montreal, QC Canada; 34 University of Saskatchewan, Regina, SK Canada; 35 L’angio-oedème héréditaire du Québec, Quebec City, QC Canada; 36 Queen’s University, Kingston, ON Canada; 37 Southern Alberta Rare Blood and Bleeding Disorders Clinic, Calgary, AB Canada

**Keywords:** Hereditary angioedema, Guideline, Recommendations, Pediatrics, Pregnancy, Lactation, Attacks, Drug therapy, Prophylaxis, Quality of life, Patient registry

## Abstract

This update to the 2019 Canadian Hereditary Angioedema (HAE) Guideline broadens its focus to include the management of patients with HAE worldwide, building on its established international framework. It has been developed through a collaboration of Canadian and international HAE experts and patient groups, coordinated by the Canadian Hereditary Angioedema Network. The objective is to provide evidence-based recommendations, using the Grading of Recommendations Assessment, Development and Evaluation system, for the management of patients with HAE. These include recommendations for the treatment of attacks, short-term prophylaxis, and long-term prophylaxis, as well as recommendations for self-administration, individualized therapy, health-related quality of life, and comprehensive care. New to the 2024 edition are specific recommendations for the treatment of angioedema attacks in individuals with HAE who are breastfeeding/lactating, as well as a dedicated section on shared decision-making. HAE results in spontaneous and often unpredictable attacks of painful swelling, typically affecting the extremities, bowel mucosa, genitals, face, and upper airway. These attacks are associated with significant functional impairment, reduced health-related quality of life, and in the case of laryngeal attacks, a high risk of mortality. Managing HAE is complex, and patient care in Canada, similar to many other countries, remains inconsistent and suboptimal. Care delivery lags behind nations that have implemented more structured management models for HAE, and offer broader access to a wider range of approved therapies. This guideline is intended to be used to optimize HAE management, highlight the importance of individualized care, and provide guidance to healthcare providers, policymakers, patients, and advocates. Primary target users include healthcare providers who are managing patients with HAE, as well as emergency and intensive care physicians, primary care physicians, gastroenterologists, dentists, otolaryngologists, pediatricians, hematologists, dermatologists, and gynecologists who will encounter patients with HAE and need to be aware of this condition. Hospital administrators, insurers and policy makers may also find this guideline helpful.

**Supplementary Information:**

The online version contains supplementary material available at 10.1186/s13223-025-00999-8.

## Background

Hereditary angioedema (HAE) is a rare autosomal dominant condition that results in spontaneous and often unpredictable attacks of painful swelling typically affecting the extremities, bowel mucosa, genitals, face, and upper airway [1, 2]. Attacks are associated with significant functional impairment, decreased health-related quality of life (HRQoL), and in the case of laryngeal attacks, mortality [2, 3]. If untreated, attacks can last 2–5 days.

HAE can be categorized into HAE due to complement 1 esterase inhibitor (*C1INH*) deficiency or HAE with normal *C1INH* (HAE-nC1INH; Table 1). HAE due to *C1INH* deficiency is caused by mutations in the serpin family G member 1 gene (*SERPING1*) encoding *C1INH*, which lead to a quantitative deficiency in *C1INH* (HAE-C1INH-Type1) or dysfunctional *C1INH* (HAE-C1INH-Type2) [2, 4]. These subtypes have a combined estimated prevalence of approximately 1:50,000, with about 25% of patients having no family history due to the occurrence of a de novo mutation [5, 6]. HAE-C1INH-Type1 is the most prevalent subtype, representing approximately 80%–85% of cases, and resulting from low antigenic and functional levels of C1INH. HAE-C1INH-Type2 accounts for approximately 15%–20% of cases and is due to impaired C1INH function, despite a normal or raised C1INH protein concentration [7, 8]. Complement component 4 (C4) is reduced in most cases of HAE due to *C1INH* deficiency and is typically low during attacks [7]. However, the sensitivity of low C4 in asymptomatic patients varies, indicating that normal C4 does not exclude diagnosis [8]. Swelling in HAE due to *C1INH* deficiency results from impaired regulation of bradykinin synthesis [9]. Plasma kallikrein cleaves high-molecular-weight kininogen to release bradykinin, a nonapeptide kinin. Bradykinin is a powerful vasodilator that increases capillary permeability, constricts smooth muscle, and stimulates pain receptors [5, 6].

**Table 1:** Laboratory findings in HAE [14]

Type	C4	C1INH antigen	C1INH function
HAE due to *C1INH* deficiency			
HAE-C1INH-Type1	↓	↓	↓
HAE-C1INH-Type2	↓	Normal or ↑	↓
HAE-nC1INH^*^	Normal	Normal	Normal

^*^Coagulation factor XII, plasminogen, angiopoietin 1, kininogen 1, myoferlin, heparan sulfate-glucosamine 3-O-sulfotransferase 6, or unknown variant. C1INH, complement 1 esterase inhibitor; C4, complement component 4; HAE, hereditary angioedema; n, normal.

In contrast to HAE due to *C1INH* deficiency, the true prevalence of HAE-nC1INH is unknown, with estimates varying widely [4, 10–12]. HAE-nC1INH is an umbrella term that encompasses several distinct subtypes of HAE, of which none are associated with mutations in *SERPING1* [4]. It is more difficult to accurately diagnose patients with HAE-nC1INH due to normal laboratory values and the numerous criteria needed to confirm the diagnosis, with confirmatory genetic testing used in only about half of cases [10]. Although HAE-nC1INH presents with similar clinical features to HAE due to *C1INH* deficiency [13], its pathogenesis has not been fully elucidated, with many individuals classified as having HAE-nC1INH with unknown cause/pathogenesis [14, 15]. To date, mutations in the following six genes have been associated with HAE-nC1INH: factor XII (*F12*), plasminogen (*PLG*), angiopoietin 1 (*ANGPT1*), kininogen 1 (*KNG1*), myoferlin (*MYOF*), and heparan sulfate-glucosamine 3-O-sulfotransferase 6 (*HS3ST6*) [16–22]*.* Two further variants, carboxypeptidase N (*CPN1*), and DAB2 interacting protein (*DAB2IP*), may result in HAE-nC1INH, but this is yet to be confirmed [23, 24]. Four distinct variants in the gene coding for coagulation factor XII (FXII) can lead to HAE-FXII [22, 25], with the Thr328Lys variant being far more common. These variants create a cleavage site for plasmin, which facilitates the activation of FXII and the generation of bradykinin [26]. In HAE-PLG, the *PLG* gene, which encodes for plasminogen, is affected. Although the full pathogenic mechanism is not completely understood, evidence suggests that plasmin derived from mutated plasminogen can directly cleave high- and low-molecular-weight kininogens to release bradykinin, independently of plasma kallikrein and activated FXII activity [19, 27, 28]. The pathogenic mechanisms underlying the remaining variants are less well understood. Variants in the *ANGPT1* gene in HAE-ANGPT1 are thought to impair the ability of the ANGPT1 protein to effectively bind its receptor, tunica interna endothelial cell kinase 2. These variants may also contribute to dysregulation of vascular permeability [21]. The likely impact of variants in *KNG1* is to make kininogen susceptible to plasma cleavage [17]. A variant in *HS3ST6* appears to impair heparan sulfate synthesis, potentially altering cell surface interactions that regulate kallikrein–kinin system activity [18]. A variant in *MYOF* may involve dysregulated vascular endothelial growth factor signalling in endothelial cells, although very little is known about this subtype [16, 29]. An international consensus group published criteria for making a diagnosis of HAE-nC1INH [12], which include: (1) a history of recurrent angioedema in the absence of concomitant hives or use of medication known to cause angioedema; (2) documented normal or near-normal C4, C1INH antigen, and C1INH function; and (3) either a genetic variant associated with the disease, or a strong family history of recurrent angioedema without hives, along with documented lack of efficacy of chronic high-dose antihistamines, montelukast (unless contraindicated), and omalizumab.

Management of HAE can be divided into various, nonexclusive approaches. The aim of treating HAE attacks, also referred to as *on-demand therapy*, is to minimize the severity and duration of attacks, including potentially fatal upper airway angioedema and associated impairment of quality of life (QoL). Short-term prophylaxis (STP) refers to treatment meant to minimize the risk of an attack when exposure to a potential or known trigger is anticipated. Long-term prophylaxis (LTP) refers to ongoing treatment of HAE aimed at preventing or minimizing the overall number, frequency, and/or severity of attacks. The details of specific therapies for these treatment approaches will be discussed in the sections that follow. The *clinical considerations* following each recommendation provide a summary of the evidence behind the proposed recommendations, as well as the practical clinical context to assist physicians in managing individual patients.

### Scope and purpose

The objective of this guideline is to provide evidence-based recommendations for the management of patients with HAE, due to *C1INH* deficiency and HAE-nC1INH, in Canada and internationally. This guideline includes recommendations for the treatment of HAE attacks, STP, and LTP, as well as recommendations for self-administration, individualized therapy, QoL, and comprehensive care. New to the 2024 version of this guideline are specific recommendations for the treatment of angioedema attacks in individuals with HAE who are breastfeeding/lactating, and a section on shared decision-making.

Managing HAE can be complex, and patient care in Canada, as in many countries, remains suboptimal and inconsistent. It lags behind nations with more organized models for HAE management, and broader access to additional licensed therapeutic options [14, 15]. It is anticipated that use of this international/Canadian guideline will not only optimize the management of HAE, but also promote individualized care.

### Intended audience

The primary target users of this guideline are healthcare providers who are managing patients with HAE due to *C1INH* deficiency or HAE-nC1INH. Other healthcare providers who may use this guideline include emergency and intensive care physicians, primary care physicians, gastroenterologists, dentists, otolaryngologists, pediatricians, hematologists, dermatologists, and gynecologists who will encounter patients with HAE and need to be aware of this condition. Hospital administrators, insurers, and policy makers may also find this guideline helpful as it is based on current evidence and provides a benchmark for HAE care.

## Methods

### Committee Members, Guideline Authors, and Conference Participants

The Canadian Hereditary Angioedema Guideline Committee (“the Committee”) is a working committee under the umbrella of the Canadian Hereditary Angioedema Network/Réseau Canadien d’angioédème héréditaire (CHAEN/RCAOH) [30]. Members of the Committee included physicians who treat HAE from across Canada, as well as the President or delegate of the Canadian HAE patient organization (HAE Canada). The Committee was responsible for defining the scope and purpose of the guideline and choosing international participants, who were selected based on their relevant expertise in HAE, its management, and guideline priorities, including self-administration, individualized therapy, HRQoL, comprehensive care, HAE in pregnant patients, HAE in pediatric patients, HAE-nC1INH, and shared decision-making. Identified experts were asked to present a summary of the evidence related to areas of interest at the CHAEN/RCAOH Guideline Conference (“the Conference”). There was also representation from Angio-oedème héréditaire du Québec (AOHQ) at the Conference. Representatives from Héma-Québec and Canadian Blood Services were invited as observers. Invitations were extended to representatives of the provincial/territorial blood coordinating offices, and the global HAE patient group, HAE International, but they declined or were unable to attend.

Guideline Authors included the Committee, international experts, registered members of CHAEN, the President and delegates of HAE Canada, and the President of AOHQ. All Guideline Authors were asked to submit standard International Committee of Medical Journal Editors (ICMJE) conflict of interest forms, which were vetted by an independent reviewer for potential conflicts. “Conference Participants” refers to Guideline Authors who attended the Conference. Only those attendees were eligible to vote on the recommendations. Therefore, “We” refers specifically to the Guideline Authors who both attended and voted. (see **Recommendation development and approval** for further details).

### Identifying the evidence

A librarian conducted a systematic search using Ovid MEDLINE on April 13, 2024, based on the predefined scope as described in Appendix 1. The search was designed to identify current evidence on LTP, STP, and acute treatment of attacks in patients of any age diagnosed with HAE due to *C1INH* deficiency or HAE-nC1INH. The search was repeated on September 9, 2024 using the same search strategy to ensure that the most recent evidence was considered at the Conference held September 20–22, 2024.

Outcomes of interest included frequency or severity of attacks, symptom relief, or HRQoL measures as reported or measured by the subject or investigator. Studies were limited to English-language publications that had been published and indexed in MEDLINE since the previous search was conducted for the 2019 International/Canadian Hereditary Angioedema Guideline in November 2018 [31]. To ensure the comprehensiveness of the evidence base, the Committee was invited to suggest additional articles for consideration.

The evidence identification and selection process followed Preferred Reporting Items for Systematic Reviews and Meta-Analyses (PRISMA) 2020 principles to ensure transparency and completeness in reporting. A total of 266 unique results were identified in the search, and two reviewers (among SB, GL, SW, and VP) independently applied the predetermined inclusion criteria to the title and abstract of each result. If either reviewer indicated that a result required further consideration at the title and abstract review stage, the full text document was retrieved and reviewed by both reviewers.

Of these unique results, 59 articles were retrieved and reviewed in full text by both reviewers. Any disagreements between the reviewers were discussed electronically until consensus was reached. If necessary, a third reviewer was consulted. Fifteen relevant randomized controlled trials and 15 lower-quality studies without randomization or blinding met the inclusion criteria and were included in evidence tables. The complete search strategy, inclusion criteria, and PRISMA flow diagram are provided in Appendix 1.

### Summarizing and evaluating the evidence

Key information, such as study design, number of patients, outcome measures, adverse events, and funding source was extracted into evidence tables for each intervention from the included studies (see Additional File 1 and Additional File 2). Evidence tables were provided to the Committee and were available for reference at the Conference.

Criteria for determining the levels of evidence and strength of each recommendation were adapted from the Grading of Recommendations Assessment, Development and Evaluation (GRADE) system [32–34], and the process was based primarily on the Journal of Clinical Epidemiology’s 2011–2013 series of articles describing the GRADE methodology. The method applied here involved evaluating the quality of each study, and then evaluating the studies together, to assign a level of evidence based on the collection of studies (see Appendix Table 5). Each identified randomized controlled trial was assessed by two reviewers for quality using the Cochrane Risk of Bias 2 (RoB2) tool [35]. A third reviewer resolved any disagreements. Randomized trials were initially rated as high-level evidence, with the quality being downgraded if there was evidence of bias on the RoB2 tool or evidence of inconsistency (see Appendix Table 6). Nonrandomized, unblinded trials were considered to be low-quality evidence.

Appendix 2 provides additional detail on how quality was assessed and the criteria used to determine the strength of recommendation. The quality ratings were presented at the Conference during the discussion of draft recommendations.

### Recommendation development and approval

Content experts were assigned specific topic areas and were asked to review the evidence tables relevant to their topic and present the body of evidence for consideration at the Conference. After the summary was presented, Conference Participants were provided an opportunity to discuss the literature. Following this discussion, each draft recommendation was presented, and the group discussed the specific wording of the recommendation before voting anonymously via electronic voting to agree or disagree with the recommendation, or abstain. If 80% consensus was not reached, there was additional group discussion, the recommendation was rephrased, and a new vote was conducted. This process was conducted a maximum of three times. If 80% consensus was not reached, it was determined that the group was unable to reach consensus.

Once Conference Participants approved the phrasing of a recommendation, the guideline methodologist (VP) presented the proposed level of evidence (high, moderate, low, very low, or consensus; see Appendix Table 5 for definitions). The level of evidence was then discussed, revised if necessary, and similarly voted on as outlined above.

The suggested strength of recommendation (strong or weak) was then presented to the group. The guideline methodologist proposed a strength of recommendation based on the level of evidence, the balance between desirable and undesirable effects, values, preferences, and cost/resource considerations. The group discussed these factors before voting to accept the proposed strength of recommendation. All votes were recorded and presented in real time with the recommendations. Table 2 provides a summary of all recommendations, the level of evidence supporting each recommendation, and the strength of each recommendation.

**Table 2:** Summary of recommendations

Recommendation	Level of evidence and strength of recommendation
Diagnosis of HAE due to *C1INH* deficiency	
1. The diagnosis of HAE due to *C1INH* deficiency should be made by measuring plasma levels of C1INH antigen or C1INH function	High, Strong
2. All individuals with a positive family history of HAE should be considered to be at risk of HAE and should be screened	Consensus, Strong
Acute treatment of HAE due to *C1INH* deficiency	
3. Effective therapies should be used for the acute treatment of attacks of angioedema to reduce duration and severity of attacks. Effective therapies include ecallantide, icatibant, intravenous C1INH (plasma-derived or recombinant), and sebetralstat	High, Strong
4. Attenuated androgens and tranexamic acid should not be used for the acute treatment of attacks	Low, Strong
5. Frozen plasma could be used for acute treatment of attacks if other recommended therapies are not available	Low, Strong
6. Attacks should be treated early to reduce morbidity	Moderate, Strong
7. Attacks should be treated early to reduce mortality	Moderate, Strong
8. All attacks of angioedema involving the upper airway are medical emergencies and must be treated immediately	Low, Strong
Acute treatment in pregnant, breastfeeding, or lactating patients with HAE	
9. pdC1INH is the treatment of choice for angioedema attacks in pregnant patients with HAE due to *C1INH* deficiency	Low, Strong
10. rhC1INH can be used for treating angioedema attacks in pregnant patients with HAE due to *C1INH* deficiency if pdC1INH is not available	Low, Strong
11. Icatibant may be used to treat angioedema attacks in pregnant patients with HAE due to *C1INH* deficiency if no other treatment is available or there is no response to pdC1INH	Low, Weak
12. Ecallantide should not be used to treat attacks in pregnant patients with HAE due to *C1INH* deficiency	Consensus, Strong
13. pdC1INH is the treatment of choice for angioedema attacks in breastfeeding/lactating patients with HAE due to *C1INH* deficiency	Consensus, Strong
14. rhC1INH may be considered for treating angioedema attacks in breastfeeding/lactating patients with HAE due to *C1INH* deficiency if pdC1INH is not available	Consensus, Strong
15. Icatibant can be used to treat angioedema attacks in breastfeeding/lactating patients with HAE due to *C1INH* deficiency, but breastfeeding/lactation should be avoided for 12 h after administration	Consensus, Strong
Acute treatment of HAE in pediatric patients with HAE due to *C1INH* deficiency	
16. All pediatric patients diagnosed with HAE, including those who are symptom-free, should have access to acute treatment	Consensus, Strong
17. Intravenous pdC1INH is an effective therapy for the acute treatment of HAE attacks in pediatric patients	Moderate, Strong
18. Icatibant is an effective therapy for the acute treatment of HAE attacks in children and adolescent patients	Moderate, Strong
19. Intravenous rhC1INH is an effective therapy for the acute treatment of HAE attacks in children and adolescent patients	Moderate, Strong
20. Ecallantide is an effective therapy for the acute treatment of HAE attacks in adolescent patients	Moderate, Strong
Diagnosis of HAE with normal *C1INH*	
21. If the diagnosis of HAE-nC1INH is suspected, a referral should be made to a physician who has expertise with this condition	Consensus, Strong
Acute treatment of HAE with normal *C1INH*	
22. pdC1INH is an effective therapy for the acute treatment of attacks in patients with HAE-nC1INH	Low, Strong
23. Icatibant is an effective therapy for the acute treatment of HAE attacks in patients with HAE-nC1INH	Low, Strong
Short-term prophylaxis	
24. Short-term prophylaxis should be considered prior to known patient-specific triggers and made available for any medical, surgical, or dental procedures in patients with HAE due to *C1INH* deficiency	Low, Strong
25. HAE-specific acute treatment should be available during and after any procedures in patients with HAE due to *C1INH* deficiency	Consensus, Strong
26. Intravenous pdC1INH or rhC1INH should be used for short-term prophylaxis in patients with HAE due to *C1INH* deficiency	Low, Strong
27. Short-term prophylaxis is recommended in the case of a cesarean delivery or intrapartum instrumentation in patients with HAE due to *C1INH* deficiency. Effective acute therapy should be immediately available for all deliveries	Consensus, Strong
28. Short-term prophylaxis should be considered prior to known patient-specific triggers and made available for any medical, dental, and surgical procedures for patients with HAE-nC1INH	Consensus, Strong
Long-term prophylaxis in HAE due to *C1INH* deficiency	
29. Long-term prophylaxis should be considered at every visit taking disease activity, burden, control, and patient preference into account	Consensus, Strong
30. All patients should have a management plan, including immediate access to effective treatment for attacks, even when on prophylaxis	Low, Strong
31. Berotralstat, donidalorsen, garadacimab, lanadelumab, and pdC1INH are effective therapies for long-term prophylaxis in patients with HAE due to *C1INH* deficiency	High, Strong
32. Berotralstat, donidalorsen, garadacimab, lanadelumab, or pdC1INH could be used as first-line therapy for long-term prophylaxis in patients with HAE due to *C1INH* deficiency	Consensus, Strong
Long-term prophylaxis in pregnant, breastfeeding, or lactating patients with HAE	
33. Long-term prophylaxis should be considered in pregnant patients with HAE	Consensus, Strong
34. pdC1INH is the treatment of choice for long-term prophylaxis in pregnant patients with HAE	Low, Strong
35. Attenuated androgens should not be used in patients with HAE during pregnancy	Consensus, Strong
36. Long-term prophylaxis should be considered in patients with HAE during the breastfeeding/lactation period	Consensus, Strong
37. pdC1INH is the treatment of choice for long-term prophylaxis in breastfeeding/lactating patients with HAE	Consensus, Strong
38. Attenuated androgens should not be used in patients with HAE during the breastfeeding/lactating period	Consensus, Strong
Long-term prophylaxis in pediatric patients with HAE due to *C1INH* deficiency	
39. When long-term prophylaxis is indicated in neonates and infants (aged 0–2 years), pdC1INH is the treatment of choice	Consensus, Strong
40. When long-term prophylaxis is indicated in children and adolescents, lanadelumab and pdC1INH are effective treatments	Moderate, Strong
41. Berotralstat is an effective therapy for long-term prophylaxis in adolescent patients with HAE due to *C1INH* deficiency	Low, Strong
42. Androgens should not be used for long-term prophylaxis in pediatric patients	Moderate, Strong
Long-term prophylaxis treatment in HAE with normal *C1INH*	
43. Long-term prophylaxis may be appropriate for some patients with HAE-nC1INH. The decision to start a patient on long-term prophylaxis should be individualized between an HAE specialist and the patient	Low, Strong
Self-administration	
44. All patients with HAE should be trained on self-administration of HAE-specific therapies if they are suitable candidates. If patients cannot self-administer therapy, provisions should be made to ensure timely access to all appropriate therapies	Consensus, Strong
Approach to individualized therapy	
45. The decision to start or stop long-term prophylaxis depends on multiple factors and should be made by the patient and an HAE specialist	Consensus, Strong
Quality of life	
46. Healthcare providers should routinely assess disease control, disease activity, and quality of life in patients with HAE using validated instruments in order to optimize HAE management	Unable to reach consensus, Strong
Shared decision-making	
47. Shared decision-making should be used to create a partnership between the physician and patient focused on individualized care	Consensus, Strong
48. Payers must consider the opinion of the HAE specialist when evaluating reimbursement for treatment of patients with HAE, including HAE-nC1INH	Consensus, Strong
Comprehensive care	
49. Comprehensive care for all patients with HAE should be provided to optimize treatment and outcomes	Low, Strong
50. All patients with HAE should be informed about HAE patient associations	Consensus, Strong
Registries	
51. Physicians should actively participate and encourage patients to enrol in an HAE registry	Consensus, Strong

Group discussions were recorded for each topic area and used to inform the clinical considerations for each recommendation.

Prior to the Conference, the Committee determined that open discussion amongst Conference Participants regarding an approach to individualized therapy would be beneficial. For this topic, small roundtable discussions were facilitated, and additional clinical considerations were evaluated prior to recommendation review and voting.

## Guideline recommendations

### *Diagnosis* of HAE due to *C1INH* deficiency

#### *Background*

The consequences of undiagnosed HAE can be severe. One study demonstrated a mortality rate of 31.4% for undiagnosed HAE patients (n = 63/201), compared with 1.33% in diagnosed patients (*n* = 7/527) [1]. Without an accurate diagnosis, patients may be unable to access appropriate medications to prevent morbidity and mortality, and may be subject to stigmatization because of their symptoms. The diagnosis of HAE due to *C1INH* deficiency should be based on a thorough history, clinical features supportive of the disease, and confirmatory laboratory investigations. HAE should be suspected in patients who have recurrent angioedema as well as in patients who have recurrent abdominal pain for which no cause is identified, particularly if there is a family history. Healthcare providers should keep in mind that the use of medications known to cause angioedema, such as angiotensin-converting enzyme (ACE) inhibitors, oral contraceptives, dipeptidyl peptidase IV (DPP-IV) inhibitors, and neprilysin inhibitors does not preclude a diagnosis of HAE, as these medications are also associated with angioedema attacks in affected individuals and may, in fact, unmask the condition.

Measurement of plasma C4 is a useful screening tool for use in individuals suspected of having HAE due to *C1INH* deficiency, since most of those affected have a reduced C4 level between attacks [5] and nearly all have a low level during attacks [7, 8]. However, C4 levels can appear normal because current assays detect both functional and nonfunctional C4 [36]. The Conference Participants asserted that a diagnosis of HAE due to *C1INH* deficiency should be made by assessing antigenic C1INH levels (low in HAE-C1INH-Type1 but normal/elevated in HAE-C1INH-Type2) or functional C1INH levels (low in both subtypes; see Table 1). If antigenic C1INH levels are investigated in isolation and found to be normal, further laboratory investigations are warranted to avoid missing a diagnosis of HAE-C1INH-Type2. Results should be confirmed with duplicate laboratory investigations after initial testing. However, HAE-specific treatment should not be delayed while awaiting confirmatory testing.

The Committee affirmed that the diagnosis and management of acquired *C1INH* deficiency is not a specific focus of this guideline.

Recommendation 1 **The diagnosis of HAE due to C1INH deficiency should be made by measuring plasma levels of C1INH antigen or C1INH function.**

**Level of Evidence: High** (83.0% Agree, 17.0% Disagree)

**Strength of Recommendation: Strong** (95.0% Agree, 2.5% Disagree, 2.5% Abstain)

#### Clinical considerations

There was extensive discussion as to which laboratory investigations should be used to confirm a diagnosis of HAE due to *C1INH* deficiency. The Conference Participants (ie, Guideline Authors who attended the Conference in person) were determined to establish a recommendation that would be applicable to the broadest audience, given that the availability of diagnostic assays can vary considerably around the world. Although assays to measure C4 are widely available and relatively inexpensive, C4 level alone should not be used to confirm or rule out a diagnosis of HAE due to *C1INH* deficiency because the assay may detect nonfunctional C4 [8, 14, 15, 36]. The Conference Participants agreed that, in most situations, a diagnosis should be made by measuring plasma levels of C1INH antigen and/or C1INH function. It was noted that an isolated finding of normal levels of C1INH antigen would require further investigation to avoid missing a diagnosis of HAE-C1INH-Type2. In some regions, *SERPING1* gene sequencing is more accessible and feasible than C1INH testing and therefore used directly for HAE evaluation [37, 38]. Sequencing *SERPING1* for pathogenic variants may be used as an alternative diagnostic approach if C1INH assays are not available or when diagnosis is uncertain based on C1INH test results. However, it is important to remember that genetic reports must be interpreted within the context of the reference laboratory’s limitations. Potential false negatives may occur in cases of large deletions, balanced translocations, or variants in the promoter region, which may not be detected by standard sequencing methods [39–41]. Although not widely available, point-of-care assays using dried blood spot to quantify C1INH function and identify *SERPING1* variants may provide the impetus needed to improve diagnostic options in locations where access to testing is currently lacking [42–44].

When diagnosing HAE due to *C1INH* deficiency in pregnancy, serum C1INH testing should be interpreted with care, as C1INH antigen levels may be transiently reduced in pregnancy and normalize postpartum [45]. However, these reductions may not be sufficient to meet the diagnostic threshold for HAE due to *C1INH* deficiency. Repeat testing is recommended postpartum to confirm the diagnosis.

Diagnosis in infants can also pose challenges. The C1INH concentration in umbilical cord blood of healthy neonates is usually lower than that of the normal adult value [46]. Interpretation of C1INH levels and function may lead to inaccurate diagnosis in infants aged < 1 year. As such, C1INH test results should be repeated after 1 year of age [47]. Given the diagnostic uncertainty of biochemical tests in very young children, genetic testing might be a useful option for determining whether a child has inherited HAE due to *C1INH* deficiency provided the variant in the affected parent is known.

Recommendation 2 **All individuals with a positive family history of HAE should be considered to be at risk of HAE and should be screened.**

**Level of Evidence: Consensus** (97.8% Agree, 2.2% Abstain)

**Strength of Recommendation: Strong** (97.8% Agree, 2.2% Disagree)

#### Clinical considerations

HAE is an autosomal dominant disease, which gives every offspring of an individual with HAE a 50% chance of inheriting the disease [48]. Whether the phenotype of recurrent angioedema is expressed depends on penetrance of the underlying genetic variant. In addition, a negative family history does not rule out the possibility that an individual has HAE. Up to 25% of cases are caused by de novo genetic variants [6, 49], meaning that many individuals may be affected without a family history of HAE.

Principles of shared decision-making should be used to ensure at-risk individuals, and their first-degree family members have the necessary information to make informed decisions about screening for HAE (see **Recommendation 47**). Family members should be screened as early as possible, with laboratory assessments repeated in individuals aged ≥ 1 year for the reasons stated previously.

### Acute treatment of HAE due to *C1INH* deficiency

#### Background

Attacks of HAE may be spontaneous or precipitated by external stimuli and range from mild to life-threatening. The decision to treat an attack depends on many variables, and the severity of an attack cannot always be predicted by its earliest manifestations. The aim of treating an attack is to reduce its duration and severity, minimize its impact on the functional ability of the patient, and reduce morbidity and potential mortality.

Despite the increase in available efficacious therapies, some therapies, which have not been demonstrated to be effective in trials for individuals with HAE, continue to be used to treat attacks due to either historical precedent or lack of awareness. It is vital to continue emphasizing that healthcare professionals use only effective, evidence-supported therapies to treat HAE, and specifically avoid therapies that lack supporting evidence, such as antihistamines, corticosteroids, or epinephrine, which treat mast cell–mediated but not bradykinin-mediated angioedema. Patients with HAE due to *C1INH* deficiency should discontinue and avoid any known medication triggers, such as estrogen-containing oral contraceptives or replacement therapy, ACE inhibitors, DPP-IV inhibitors, and neprilysin inhibitors [50–52].

Recommendation 3 **Effective therapies should be used for the acute treatment of attacks of angioedema to reduce duration and severity of attacks. Effective therapies include ecallantide, icatibant, intravenous C1INH (plasma-derived or recombinant), and sebetralstat.**

**Level of Evidence: High** (95.2% Agree, 4.8% Disagree)

**Strength of Recommendation: Strong** (100% Agree)

#### Clinical considerations

Several effective therapies are available for the acute treatment of HAE attacks. Table 3 lists the specific agents, with their mechanism of action, licensed indications internationally, and recommended dosages. The following therapies are discussed in alphabetical order, without implying any hierarchy of preference. Given the variation in patient response to acute therapies [53], it is crucial that healthcare professionals have access to another effective acute treatment, if necessary, to optimize patient outcomes. All patients should have access to at least two doses of acute therapy, and their ability to administer these therapies should be routinely assessed. Effective treatment for attacks should not only relieve symptoms but also provide patients with enough time to safely access urgent care if needed.

**Table 3:** Therapies for HAE supported by high-level evidence

HAE-specific treatment	Product name	Company	Mechanism of action	Route of admin	Dose	Age indications and licensed countries
*Acute treatment*						
pdC1INH	Berinert^®a^	CSL	Replaces C1INH	IV	20 IU/kg	No age restriction: Australia [68], Canada [69], EU [70], UK [71, 72], US [67] Approved (age criteria not specified): Japan [73]
pdC1INH	Cinryze^®^^b,c^	Takeda	Replaces C1INH	IV	Adults and adolescents ≥ 12 years: 1000 IU Children 2–11 years: > 25 kg: 1000 IU 10–25 kg: 500 IU	Adults, adolescents, and children ≥ 2 years: EU [74], UK [75]
Ecallantide	Kalbitor^®^	Takeda	Selective, reversible inhibitor of plasma kallikrein	SC	30 mg (3 × 10 mg/1 mL)	Adults and adolescents ≥ 12 years: US [54]
Icatibant	Firazyr^®d,e^	Takeda	Synthetic selective and specific antagonist of bradykinin B2 receptor	SC	Adults: 30 mg Adolescents and children 2–17 years and weighing < 65 kg: dose adjusted based on body weight	Adults, adolescents, and children ≥ 2 years: Australia [58], Canada [57], EU [59], Japan [61], UK [60] Adults only: US [62]
rhC1INH (conestat alfa)	Ruconest^®^	Pharming	Replaces C1INH	IV	Body weight: < 84 kg: 50 IU/kg ≥ 84 kg: 4200 IU	Adults, adolescents, and children ≥ 2 years: EU [80], UK [79] Adults and adolescents ≥ 12 years: US [81]
Sebetralstat	Ekterly^®f^	KalVista	Plasma kallikrein inhibitor	Oral	600 mg (2 × 300 mg tablets)	Adults and adolescents ≥ 12 years: US [91]
Sebetralstat	Ekterly^®f^	KalVista	Plasma kallikrein inhibitor	Oral	300 mg	Adults and adolescents ≥ 12 years: EU [154, 155], UK [90]
*Pre-procedural*						
pdC1INH	Berinert^®^^a^	CSL	Replaces C1INH	IV	Adult: 1000 IU Pediatric: 15–30 IU/kg	No age restriction: EU [70], UK [71, 72] Approved (age criteria not specified): Japan [73]
pdC1INH	Cinryze^®b^	Takeda	Replaces C1INH	IV	≥ 12 years: 1000 IU 2–11 years: > 25 kg: 1000 IU 10–25 kg: 500 IU	Adults, adolescents, and children ≥ 2 years: EU [74], UK [75]
*Long-term prophylaxis*						
pdC1INH	Cinryze^®b,g^	Takeda	Replaces C1INH	IV	≥ 12 years: 1000 IU every 3–4 days 6–11 years: 500 IU every 3–4 days	Adults, adolescents, and children ≥ 6 years: EU [74], UK [75], US [140] Adults and adolescents: Canada [141]
pdC1INH	Haegarda^®h^	CSL	Replaces C1INH	SC	60 IU/kg twice-weekly (every 3–4 days)	No age restriction: Japan [270] Adults and children ≥ 6 years: US [253] Adults and children ≥ 8 years: Australia [271] Adults and adolescents: Canada [192], EU [272], UK [273, 274]
Berotralstat	Orladeyo^®^	BioCryst	Plasma kallikrein inhibitor	Oral	150-mg capsule once-daily	Adults and adolescents ≥ 12 years: Japan [199], US [198] Adults and adolescents ≥ 12 years and weighing ≥ 40 kg: Canada [193], EU [200], UK [201]
Donidalorsen	Dawnzera™	Ionis/Otsuka	Targets prekallikrein RNA	SC	80 mg every 4 or 8 weeks	Adults and adolescents ≥ 12 years: US [209]
Garadacimab	Andembry^®i^	CSL	FXIIa-inhibitory monoclonal antibody	SC with auto-injector	Loading dose: 2 × 200 mg Maintenance dose: 200 mg monthly, starting one month after loading dose	Adults and adolescents ≥ 12 years: Australia [211], Canada [216], EU [212], Japan [214], US [215], UK [213]
Lanadelumab	Takhzyro^®j,k^	Takeda	Fully human monoclonal antibody that binds plasma kallikrein and inhibits its proteolytic activity	SC	≥ 12 years: 300 mg every 2 weeks ≥ 2 to < 12 years: dose adjusted	Adults, adolescents, and children ≥ 2 years: Australia [219], EU [220], UK [221], US [218] Adults and adolescents ≥ 12 years: Canada [191], Japan [217]

Please refer to current country-specific monographs for further details regarding specific indications and listings of adverse events. ^a^Marketed as Berinert IV in Australia [68]; Berinert 500/1500 in Canada [69], EU [70], and UK [71, 72]; Berinert P I.V. Injection 500 in Japan [73]. ^b^Approved but not marketed in Australia since no financial agreement was reached [355]. ^c^A second dose may be administered if the patient has not responded adequately after 60 min (or sooner in the case of laryngeal attacks) [74]. ^d^In case of insufficient relief or recurrence of symptoms in adults, a second injection of icatibant can be administered after 6 h. If the second injection produces insufficient relief or a recurrence of symptoms is observed, a third injection can be administered after a further 6 h. No more than three injections of icatibant should be administered in a 24-h period [57, 58]. ^e^Dosage regimen for pediatric patients: 12–25 kg body weight, 10 mg dose (1.0 mL injection volume); 26–40 kg: 15 mg (1.5 mL); 41–50 kg: 20 mg (2.0 mL); 51–65 kg: 25 mg (2.5 mL); > 65 kg: 30 mg (3.0 mL) [57–59]. ^f^First dose is taken orally at the earliest recognition of an HAE attack. A second dose of 600 mg (two tablets) may be taken 3 h after the first dose if response is inadequate, or if symptoms worsen or recur. Maximum recommended dosage: 1,200 mg in any 24-h period. ^g^EU/UK: The dosing interval and dose of pdC1-INH for prophylaxis may need to be adjusted according to individual response [74, 75]. US: For patients who have not responded adequately, doses up to 2000 IU (not exceeding 80 IU/kg) every 3 or 4 days may be considered for adults and adolescents (≥ 12 years), and up to 1000 IU every 3 or 4 days for pediatric patients (6–11 years), based on individual patient response [75]. ^h^Marketed as Berinert SC in Australia [271], Berinert 2000/3000 in EU [272], and Berinert S.C. Injection 2000 in Japan [270]. ^i^The recommended dose of garadacimab is an initial loading dose of 400 mg administered subcutaneously as two 200-mg injections on the first day of treatment, followed by a monthly dose of 200 mg [211, 212]. ^j^Canada/Japan/US: A dosing interval of 300 mg lanadelumab every 4 weeks may be considered if the patient is well-controlled (eg, attack-free) for more than 6 months [191, 217, 218]. Australia/EU/UK: In adults who are stably attack-free on treatment, a dose reduction to 300 mg lanadelumab every 4 weeks may be considered, especially in patients with low weight [219–221]. EU/UK: In adults and adolescents (12 to < 18 years) with a body weight less than 40 kg, a starting dose of 150 mg lanadelumab every 2 weeks may also be considered. In patients who are stably attack free on treatment, a dose reduction to 150 mg lanadelumab every 4 weeks may be considered [220, 221]. ^k^EU/UK pediatric dose adjustment: 10 to < 20 kg, 150 mg every 4 weeks, 20 to < 40 kg, 150 mg every 2 weeks; ≥ 40 kg, 300 mg every 2 weeks [220, 221]. Australia/US pediatric dose adjustment: aged 2 to < 6 years, 150 mg every 4 weeks; aged 6 to < 12 years: 150 mg every 2 weeks [218, 219]. admin, administration; C1INH, C1 esterase inhibitor; EU, European Union; FXIIa, activated factor XII; HAE, hereditary angioedema; IU, international units; IV, intravenous; pd, plasma derived; rh, recombinant human; SC, subcutaneous; UK, United Kingdom; US, United States.

Plasma kallikrein generates bradykinin through cleavage of high-molecular-weight kininogen [9]. Ecallantide (Kalbitor^®^) is a 60-amino acid recombinant protein that selectively and reversibly inhibits kallikrein. Ecallantide is mainly available in the United States (US), with limited availability reported in other regions. It is administered as three 10-mg subcutaneous (SC) injections for a total dose of 30 mg [54]. It has been shown to effectively treat attacks in adolescent and adult patients with HAE due to *C1INH* deficiency [55]. Hypersensitivity reactions have been described with this agent in 5% of administrations, of which approximately 50% were possible anaphylactic reactions [56]. It should only be administered by a healthcare practitioner in a location where anaphylaxis can be appropriately managed [54].

Bradykinin is a key mediator in inducing angioedema through activation of the bradykinin B2 receptor [9]. Icatibant (Firazyr^®^) is a synthetic decapeptide and a competitive antagonist selective for the bradykinin B2 receptor [57]. It is approved for acute treatment in patients with HAE aged 2 years and older in Australia [58], Canada [57], Europe [59, 60], and Japan [61]. It is approved only in adults in the US [62]. In adults, the recommended dose is a single 30-mg SC injection. Additional doses may be administered every 6 h if needed, with a maximum of three doses in 24 h. In children aged ≥ 2 years, the recommended dose is a single 10–25 mg SC injection based on body weight. It is generally systemically well tolerated, although 97% of patients experience injection-site reactions, which could include transient local pain, swelling, and erythema at the injection site. The efficacy and safety of icatibant in routine clinical practice have been well established in phase 3 trials [63, 64], and are supported by more than 10 years of data from the Icatibant Outcome Survey [65].

Plasma-derived C1INH (pdC1INH, Berinert^®^ or Cinryze^®^) is a human blood product with a long history of use. Treatment with pdC1INH replaces the deficient protein in patients with HAE due to *C1INH* deficiency. It has been shown to effectively treat attacks in adults and children with HAE due to *C1INH* deficiency [66]. It is administered intravenously either by healthcare professionals or by patients or caregivers who have been trained in its administration [67].

pdC1INH (Berinert^®^) is approved in Australia [68], Canada [69], Europe [70–72], Japan [73], and the US [67] for the treatment of HAE attacks, with no age restriction in most of these regions. pdC1INH (Cinryze^®^) is approved in Europe for acute treatment in patients aged ≥ 2 years [74, 75]. The recommended dose for acute treatment of attacks is derived from clinical trials. Since there have been no head-to-head trials comparing pdC1INH products, it cannot be concluded that these products have equivalent efficacy. However, there is no evidence suggesting a difference in efficacy, and a shared biological mechanism supports a rationale for similar effectiveness. The pdC1INH products are safe and well tolerated when used as indicated, with no documented transmission of infectious agents [76]. pdC1INH continues to play an important role in the treatment of attacks in patients with HAE due to *C1INH* deficiency [77], despite the approval of more recent treatment options.

Recombinant human C1INH (rhC1INH [conestat alfa; Ruconest^®^]) is generated in the mammary glands of transgenic rabbits and is identical to pdC1INH except for the degree of protein glycosylation [78–81]. This difference in glycosylation results in shorter plasma mean half-life of the recombinant product [82, 83]; however, the impact of this on physiological activity remains unknown [84]. rhC1INH has been shown to effectively treat attacks in patients with HAE due to *C1INH* deficiency aged 2 years and older [85, 86]. It is approved for use in patients aged ≥ 2 years in Europe [79, 80] and in patients ≥ 12 years in the US [81]. The recommended dose is 50 international units (IU)/kg with a maximum of 4200 IU administered as a slow intravenous (IV) injection. Although modern manufacturing and purification processes have reduced rabbit protein content in rhC1INH to very low levels (< 0.002% host-related impurities), its use remains contraindicated in individuals with a known or suspected rabbit allergy due to the potential for anaphylactic reactions [80, 81, 87].

Two randomized controlled trials evaluated sebetralstat (Ekterly^®^), an oral kallikrein inhibitor, as on-demand therapy for HAE attacks in patients with HAE due to *C1INH* deficiency [88, 89]. A phase 2 trial in 68 adults (≥ 18 years) not receiving LTP and with ≥ 3 attacks in the prior 3 months showed that a single 600 mg dose of sebetralstat significantly improved time to conventional treatment (8.0 h vs. > 12 h; *P* = 0.0010) [88]. Fewer attacks required conventional treatment at 12 h (15% vs. 21%; *P* = 0.006) and 24 h (30% vs. 40%; *P* = 0.034) with sebetralstat. The phase 3 KONFIDENT trial in 110 participants (≥ 12 years) with ≥ 2 recent attacks included patients with or without LTP, and showed faster symptom relief with 300 mg and 600 mg sebetralstat versus placebo (median 1.61 h and 1.79 h vs. 6.72 h; *P* < 0.001 and *P* = 0.001, respectively) [89]. Time to reduction in severity was also shorter (9.27 h and 7.75 h vs. > 12 h; *P* = 0.004 and *P* = 0.003), and more attacks fully resolved within 24 h (42.5% and 49.5% vs. 27.4%). Prespecified subgroup analyses demonstrated consistent efficacy regardless of LTP use. We (the subset of Guideline Authors who attended the Conference and participated in voting on recommendations) assessed the body of evidence for sebetralstat as high quality. This assessment was based on individual study evaluations using the Cochrane RoB2 tool (see Additional File 1) and the consistent therapeutic effects observed across studies, including faster symptom relief. Based on the level of evidence (high), the relevance of the outcomes to patients, and the low risk of treatment-related adverse events, we voted for a strong recommendation in favour of the use of sebetralstat for the acute treatment of attacks. Since the Conference, sebetralstat has been approved has been approved as an orally administered acute treatment for HAE attacks in adults and adolescents ≥ 12 years in the UK [90], the US [91], and Europe (following a positive opinion from the European Committee for Medicinal Products for Human Use [92]).

We took the additional step to consolidate the 2019 recommendations for all effective therapies supported by high-level evidence and with a strong recommendation in favour of use. No other randomized controlled trial data were retrieved in the systematic search; however, a prospective real-world study evaluating the efficacy and safety of a rhC1INH was identified (see Additional File 2).

Recommendation 4 **Attenuated androgens and tranexamic acid should not be used for the acute treatment of attacks.**

**Level of Evidence: Low** (97.8% Agree, 2.2% Disagree)

**Strength of Recommendation: Strong** (95.3% Agree, 4.7% Disagree)

#### Clinical considerations

Attenuated androgens, such as the 17 α-alkylated anabolic androgen danazol, and antifibrinolytic drugs, such as tranexamic acid, have not been shown to be efficacious in the acute treatment of attacks of HAE due to *C1INH* deficiency. Given the lack of evidence for these agents in the acute treatment of HAE, we strongly agreed that they should not be used for the treatment of acute HAE attacks.

Recommendation 5 **Frozen plasma could be used for acute treatment of attacks if other recommended therapies are not available.**

**Level of Evidence: Low** (100% Agree)

**Strength of Recommendation: Strong** (94.8% Agree, 5.1% Disagree)

#### Clinical considerations

Frozen plasma (FP) is a blood product containing C1INH along with other plasma proteins. Compared with solvent/detergent-treated plasma (SDP), FP is less safe due to lower pathogen inactivation. There is low level of evidence that FP is effective in the acute treatment of attacks of HAE due to *C1INH* deficiency. Additionally, FP contains substrates that may promote bradykinin generation, potentially worsening angioedema attacks. Both FP and SDP are only available for in-hospital use, and there can be significant delays—sometimes up to 24 h—in obtaining these products [93, 94]. We continue to strongly believe that FP products be used only when other approved HAE-specific therapies are unavailable. Every effort should be made to provide timely, appropriate HAE-specific therapy for HAE attacks (see Table 3). The decision to use FP must consider local resources and individual patient factors, weighing the risks and benefits of FP treatment versus symptomatic relief only. Therefore, FP use should be limited to life-threatening or severely painful attacks when no other HAE-specific therapies are accessible.

Recommendation 6 **Attacks should be treated early to reduce morbidity.**

**Level of Evidence: Moderate** (97.8% Agree, 2.2% Abstain)

**Strength of Recommendation: Strong** (100% Agree)

#### Clinical considerations

Early treatment leads to more rapid symptom resolution. Although high-level evidence is lacking, multiple observational studies have suggested that initiating treatment early can shorten the duration of HAE attacks in some patients [66, 95–100]. In individuals who experience a clearly identifiable prodromal phase, beginning therapy during prodromal symptoms may be as effective in preventing progression to an attack as treating after attack onset [101]. Therefore, despite the absence of high-level evidence, we strongly endorsed early treatment to reduce morbidity and potentially mitigate overall attack severity [102]. To help limit the potential barriers to accessing therapy in a timely manner, patients should be trained on how to self-administer therapies appropriate for the acute treatment of HAE attacks (see **Recommendation 44**). If patients cannot self-administer, efforts should be made to ensure that therapy is made available to them without a significant delay.

Recommendation 7 **Attacks should be treated early to reduce mortality.**

**Level of Evidence: Moderate** (100% Agree)

**Strength of Recommendation: Strong** (100% Agree)

#### Clinical considerations

The historical mortality rate of HAE due to *C1INH* deficiency was approximately 30% before the advent of effective therapies [103]. As described in the section on **Diagnosis of HAE due to *C1INH* deficiency,** mortality rates are several fold higher in undiagnosed versus diagnosed individuals (31.4% vs. 1.33%) [1]. Since deaths continue to occur when diagnosis is delayed and/or when effective HAE therapies are not used [104–108], it can be assumed that early treatment of an HAE attack reduces mortality. Despite the absence of a high level of evidence, we strongly endorsed early treatment with effective therapies in an attempt to reduce mortality. In the coming years, evidence from prospective registry data collection (eg, the Angioedema Centers of Reference and Excellence [ACARE] Registry; the Canadian HAE Registry; the United States HAE Association Scientific Registry) may shed more light on the impact of HAE therapies on morbidity and mortality [13].

Recommendation 8 **All attacks of angioedema involving the upper airway are medical emergencies and must be treated immediately.**

**Level of Evidence: Low** (100% Agree

**Strength of Recommendation: Strong** (100% Agree)

#### Clinical considerations

Attacks of HAE are unpredictable and potentially life-threatening. Mortality due to laryngeal angioedema is well recognized [1]. All attacks of laryngeal angioedema should be considered medical emergencies, and effective HAE therapies should be readily available and given immediately. It is also recommended that all patients with laryngeal edema, even following self-therapy, be assessed in the emergency department, in case the angioedema does not respond to therapy and expertise in airway management is required [109].

### Acute treatment in pregnant, breastfeeding, or lactating patients with HAE

#### Background

The gynecologic and obstetrical management of patients with HAE due to *C1INH* deficiency presents a unique challenge to healthcare providers. The impact of pregnancy on HAE symptoms is uncertain and may vary from pregnancy to pregnancy [89]. Pregnancy may worsen, improve, or have no effect on angioedema attacks [110–113]. At present, it is not clear whether symptoms tend to be more severe in any specific trimester [112, 114].

Vaginal delivery does not appear to be a trigger [110–113, 115], which suggests that women can forego STP for an anticipated uncomplicated vaginal delivery, although acute treatment should always be readily available. STP is recommended for cesarean delivery or intrapartum instrumentation (see section on **Short-term prophylaxis**) [111]. There is significant risk of attacks in the postpartum period, and some individuals may experience increased attack frequency during breastfeeding. This risk should be anticipated so that it can be treated should an attack occur.

Pregnant patients with HAE should be closely monitored by an HAE expert in conjunction with a team of relevant medical professionals, with acute treatment for angioedema attacks selected on an individual basis. The medication dosage is similar to that used in patients with HAE who are not pregnant, despite there being limited data supporting therapeutic options during pregnancy [14, 111, 116]. Due to ethical reasons, there are no randomized controlled trial data assessing the efficacy and safety of medications used to treat attacks in pregnant patients with HAE, although many cases have been documented [112, 114, 117–126]. The Guideline Authors recommended that healthcare professionals enrol pregnant patients with HAE in established registries to contribute to the evidence based on treatment outcomes during pregnancy.

The Committee affirmed that breastfeeding/lactation in patients with HAE would be specifically addressed in this guideline.

Recommendation 9 **pdC1INH is the treatment of choice for angioedema attacks in pregnant patients with HAE due to C1INH deficiency.**

**Level of Evidence: Low **(100% Agree)

**Strength of Recommendation: Strong** (100% Agree)

#### Clinical considerations

We unanimously agreed that there was low-level evidence supporting the use of IV pdC1INH to treat acute HAE attacks in pregnancy, based on evidence-based practice guidelines [14, 15, 111], retrospective studies [112–114, 127, 128], and case reports [129, 130]. Despite the low level of evidence, we strongly endorsed pdC1INH as the treatment of choice to reduce morbidity and mortality in pregnant patients with HAE due to *C1INH* deficiency. Similarly, we strongly recommended using pdC1INH for angioedema attacks in pregnant patients with HAE-nC1INH, despite the lack of well-controlled studies in this patient population. This therapy is cited by other guidelines as the preferred first-line treatment option during pregnancy [14, 15]. Furthermore, IV pdC1INH has the fewest reported side effects of HAE treatments and has a long history of safe use during pregnancy, despite the lack of approval by any regulatory agency for use in pregnant patients with HAE [126]. The licensed indications and recommended dosing of IV pdC1INH for the acute treatment of attacks are listed in Table 3.

Recommendation 10 **rhC1INH can be used for treating angioedema attacks in pregnant patients with HAE due to C1INH deficiency if pdC1INH is not available.**

**Level of Evidence: Low** (100% Agree)

**Strength of Recommendation: Strong** (97.7% Agree, 2.3% Abstain)

#### Clinical considerations

Intravenous rhC1INH may be considered for life-threatening HAE attacks during pregnancy when pdC1INH is unavailable, has been ineffective for the patient, or is preferred over a plasma-derived product by the patient. The evidence for rhC1INH in pregnant patients with HAE is derived from case series and case reports [118–120]. Reproductive toxicity was observed in one animal study [80]; however, no adverse effects were observed with rhC1INH use in pregnant patients in the limited post-marketing safety data available [81]. Therefore, rhC1INH should only be considered during pregnancy if the treating physician deems the benefits to outweigh the potential risks, or the patient expresses a strong personal preference for rhC1INH, given the absence of adequate and well-controlled studies.

Recommendation 11 **Icatibant may be used to treat angioedema attacks in pregnant patients with HAE due to C1INH deficiency if no other treatment is available or there is no response to pdC1INH.**

**Level of Evidence: Low** (95.4% Agree, 2.3% Disagree, 2.3% Abstain)

**Strength of Recommendation: Weak** (84.1% Agree, 15.9% Disagree)

#### Clinical considerations

Subcutaneous icatibant may be used in the case of life-threatening attacks during pregnancy when no other treatment is available or when pdC1INH or rhC1INH has proven inefficacious. Evidence on the use of icatibant in pregnancy is scarce and primarily consists of case reports with only a few treated HAE attacks [117, 119, 121–125]. Although the potential risk for pregnant patients with HAE is unknown, there is evidence of fetal harm with icatibant in animal studies [59, 62], and icatibant should be avoided once pregnancy is confirmed. The decision to use icatibant during pregnancy should be made collaboratively between the patient and their HAE specialist, through shared decision-making with careful consideration of the potential benefits and risks (see section on **Shared decision-making**).

Recommendation 12 **Ecallantide should not be used to treat attacks in pregnant patients with HAE due to C1INH deficiency.**

**Level of Evidence: Consensus** (95.0% Agree, 2.5% Disagree, 2.5% Abstain)

**Strength of Recommendation: Strong** (97.4% Agree, 2.6% Disagree)

#### Clinical considerations

There are no data on the use of SC ecallantide in pregnant patients with HAE. We agreed that ecallantide should be avoided once pregnancy is confirmed, given the risk of anaphylaxis [54].

Recommendation 13 **pdC1INH is the treatment of choice for angioedema attacks in breastfeeding/lactating patients with HAE due to C1INH deficiency.**

**Level of Evidence: Consensus** (100% Agree)

**Strength of Recommendation: Strong** (95.4% Agree, 4.6% Disagree)

Recommendation 14 **rhC1INH may be considered for treating angioedema attacks in breastfeeding/lactating patients with HAE due to C1INH deficiency if pdC1INH is not available.**

**Level of Evidence: Consensus** (100% Agree)

**Strength of Recommendation: Strong** (97.7% Agree, 2.3% Disagree)

#### Clinical considerations

Given the absence of scientific data, we unanimously agreed on consensus level of evidence for the use of pdC1INH (the treatment of choice) or rhC1INH (if pdC1INH is unavailable) to treat attacks during the breastfeeding/lactating period. Any risk to the child cannot be excluded since it is not known whether pdC1INH or rhC1INH are excreted in human milk [67, 74, 80, 81]. rhC1INH should only be considered during the breastfeeding/lactation period if the treating physician deems the benefits to outweigh the potential risks [81].

Recommendation 15 **Icatibant can be used to treat angioedema attacks in breastfeeding/lactating patients with HAE due to C1INH deficiency, but breastfeeding/lactation should be avoided for 12 hours after administration.**

**Level of Evidence: Consensus** (90.2% Agree, 4.9% Disagree, 4.9% Abstain)

**Strength of Recommendation: Strong** (85.0% Agree, 10.0% Disagree, 5.0% Abstain)

#### Clinical considerations

Given the absence of scientific data, we agreed on consensus level of evidence for the use of icatibant to treat attacks during the breastfeeding/lactating period. It is not known whether icatibant is excreted in human milk, but it is recommended that patients with HAE who use icatibant should avoid breastfeeding/using expressed milk for 12 h after treatment [59].

#### Clinical considerations

There are no data on the use of ecallantide in breastfeeding/lactating patients with HAE or on the presence of ecallantide in human milk [54]. The benefits of breastfeeding/lactation should be weighed alongside any potential harm and the clinical need to resume ecallantide [54], with a decision made collaboratively between the patient and their physician (see **Shared decision-making**).

### Acute treatment of HAE in pediatric patients with HAE due to *C1INH* deficiency

#### Background

This section focuses on acute treatment in children aged 2–12 years and adolescents aged 12–17 years with HAE due to *C1INH* deficiency, as trial data are lacking for children under 2 years and those with HAE-nC1INH. Although HAE can present at any age, it is rare in utero but has been observed in 14% of children before the age of 1 year according to a multicentre cohort of pediatric patients in Brazil [120, 131, 132]. The reported age of onset of attacks varies from 4.4 to 18 years, with the mean age at first attack of 10 years [131]. It is possible that abdominal symptoms of HAE are overlooked in infants, as young patients may be unable to describe their symptoms clearly. Abdominal discomfort and pain are common complaints in childhood, and the cause of these symptoms may easily be mistaken in this population [133–137]. In 50% of cases, the initial onset of symptoms occurs within the first 7 years of life [138].

Recommendation 16 All pediatric patients diagnosed with HAE, including those who are symptom-free, should have access to acute treatment.

**Level of Evidence: Consensus** (100% Agree)

**Strength of Recommendation: Strong** (100% Agree)

#### Clinical considerations

Symptom-free refers to patients who have been diagnosed with inherited *C1INH* deficiency but have not yet demonstrated any symptoms of the disease. According to retrospective and prospective analyses, between 16 and 33% of diagnosed pediatric patients with HAE are asymptomatic [138]. Due to the risks associated with the disease, including that upper airway obstructions occur sooner in children than in adults [139], all diagnosed patients should have ready access to acute treatment either at home or at a nearby medical facility. Administering on-demand treatment during attacks in young children presents substantial challenges, since it requires special dosages and equipment [57, 59, 69, 70, 74, 140, 141]. Additionally, managing the storage and administration of treatment in nurseries and schools adds another layer of complexity.

The following are drug-specific recommendations for the acute treatment of HAE attacks in pediatric patients.

Recommendation 17 **Intravenous pdC1INH is an effective therapy for the acute treatment of HAE attacks in paediatric patients.**

**Level of Evidence: Moderate** (100% Agree)

**Strength of Recommendation: Strong** (100% Agree)

#### Clinical considerations

When children are treated with pdC1INH for HAE attacks, responses are consistent with those of adults [142]. Studies have demonstrated that the intervention is safe and well tolerated in the pediatric population [66] and effective in reducing time to symptom relief [143–145]. Also similar to adults, data suggest that early treatment with pdC1INH in the pediatric population leads to more rapid symptom resolution [97, 100, 144]. The recommendation for IV pdC1INH remained unchanged from the previous edition of this guideline, and the Guideline Authors affirmed that its use in pediatric patients is appropriate given the lack of an age restriction for one product [67, 69, 70]. Dosing for pdC1INH is: 20 IU/kg IV Berinert^®^ regardless of age [66, 67, 69, 70, 142, 146], 500 IU IV Cinryze^®^ for children weighing 10–25 kg, and 1000 IU IV Cinryze® for children weighing > 25 kg [74, 131, 147]. (Note, the Cinryze^®^ dose is generally rounded to the nearest 500 IU).

Recommendation 18 **Icatibant is an effective therapy for the acute treatment of HAE attacks in children and adolescent patients.**

**Level of Evidence: Moderate** (100%)

**Strength of Recommendation: Strong** (100%)

#### Clinical considerations

Subcutaneous icatibant has been approved to treat patients ≥ 2 years of age in some countries (see Table 3) [91]. Depending on the age of the patient, a single SC dose of 0.4 mg/kg (to a maximum dose of 30 mg) injected into the abdomen can be self-administered, or given by a caregiver. Subcutaneous administration is advantageous as it avoids the need for IV access, which can be particularly challenging in pediatric patients [57, 59, 148]. The weight-based pediatric dosing of icatibant was evaluated over four attacks in two pediatric patients in an open-label trial in Japan [59, 149]. For each attack, a single administration of icatibant provided rapid resolution of symptoms (time to symptom resolution, 0.9–1.0 h). The reported findings were consistent with the treatment response observed in adults [63, 150]. Based on these new data, we concluded that it was appropriate to elevate the level of evidence for the recommendation to moderate. The recommendation also now specifies its use in children and adolescent patients, which aligns with the age group of participants (2–17 years) enrolled in the pivotal phase 3 pediatric study [151].

A population modelling and simulation analysis revealed that weight band–based dosing regimens (10 mg for 12–25 kg, 15 mg for 26–40 kg, 20 mg for 41–50 kg, 25 mg for 51–65 kg, and 30 mg for > 65 kg) produced icatibant exposure comparable to the 0.4-mg/kg dose [152].

Recommendation 19 **Intravenous rhC1INH is an effective therapy for the acute treatment of HAE attacks in children and adolescent patients.**

**Level of Evidence: Moderate** (100% Agree)

**Strength of Recommendation: Strong** (100% Agree)

#### Clinical considerations

rhC1INH was evaluated in 20 children aged 2–13 years with HAE due to *C1INH* deficiency in an open-label, phase 2 study [86]. A total of 73 HAE attacks were treated with IV rhC1INH at a dose of 50 IU/kg (up to 4200 IU). Most attacks (70 [95.9%]) were treated with a single dose, with the median time to onset of symptom relief (primary endpoint) of 60 min, and most children achieving symptom relief within 4 h. No serious drug-related adverse events or hypersensitivity reactions were reported. rhC1INH has been approved to treat patients ≥ 2 years of age in some countries (see Table 3) [80]. Based on these data, the Conference Participants agreed to support the new wording and level of evidence for the use of IV rhC1INH in the acute treatment of HAE attacks in children and adolescent patients.

Recommendation 20 **Ecallantide is an effective therapy for the acute treatment of HAE attacks in adolescent patients.**

**Level of Evidence: Moderate** (100% Agree)

**Strength of Recommendation: Strong** (97.6% Agree, 2.4% Disagree)

#### Clinical considerations

Ecallantide has been studied and approved for use in adolescents aged ≥ 12 years in the US based on pooled data from four clinical studies [54, 153]. This analysis of pooled data demonstrated that ecallantide is efficacious in children as young as 10 years of age. Based on these data and recommendations from other consensus guidelines [14, 15], the Conference Participants elevated the level of evidence to moderate. Ecallantide is administered under the supervision of a healthcare provider as three SC injections for a total of 30 mg [54]; it cannot be self-administered.

As described in the section on **Acute treatment of HAE due to *C1INH* deficiency**, since the Conference, sebetralstat has been approved in Europe, the UK, and US for use in patients as young as 12 years (see Table 3) [90, 91, 154, 155]. Due to limited published evidence for the adolescent subgroup, particularly regarding adverse events, the Conference Participants did not make a specific recommendation for this age group. Phase 3 studies are underway to evaluate the safety and efficacy of novel oral agents in pediatric patients. In particular, sebetralstat is being assessed in the open-label KONFIDENT-KID trial (NCT06467084) in children aged 2–11 years, with a planned enrolment of 24 patients and an estimated completion date in 2027 [156]. These data may support future regulatory considerations for use in younger children.

### Diagnosis of HAE with normal *C1INH*

#### Background

The presence of HAE-nC1INH was first reported in Canada and in Germany by Binkley and Bork respectively in 2000 [157, 158]. When the International/Canadian Hereditary Angioedema Guideline was published in 2019, HAE-nC1INH could be subdivided by causative variants affecting coagulation FXII (HAE-FXII), angiopoietin-1 (HAE-ANGPT), and plasminogen (HAE-PLG), with the remainder classified as having an unknown mutation [31]. Although the true prevalence of HAE-nC1INH remains unknown, the use of whole genome sequencing has led to identification of further associated variants in the genes encoding kininogen 1 (*KNG1*), myoferlin (*MYOF*), and heparan sulfate-glucosamine 3-O-sulfotransferase 6 (*HS3ST6)* [16–18]*.* A mutation in exon 10 of *KNG1* potentially making kininogen susceptible to plasma cleavage was identified in three consecutive generations of the same family in Germany [17]. A gain-of-function mutation in *MYOF* potentially leading to dysregulated vascular endothelial growth factor signalling was reported in four members of a multigenerational family in Italy [16]. Mutations in *HS3ST6* identified in three members of a multigenerational family in Germany appears to impair heparan sulfate synthesis, potentially altering cell surface interactions that regulate kallikrein–kinin system activity [18]. The genetic causes of HAE-nC1INH with unknown mutations will continue to be elucidated over the coming years; however, it is important to note that, aside from *F12* and *PLG*, the mechanisms discussed remain largely putative, and definitive functional studies are needed to move beyond these speculative mechanisms.

Recommendation 21 **If the diagnosis of HAE-nC1INH is suspected, a referral should be made to a physician who has expertise with this condition.**

**Level of Evidence: Consensus** (97.8% Agree, 2.2% Disagree)

**Strength of Recommendation: Strong** (97.6% Agree, 2.4% Disagree)

#### Clinical considerations

We recommended that the diagnosis of HAE-nC1INH be based on clinical evaluation by an expert physician. The current criteria for the diagnosis of HAE-nC1INH (based on consensus recommendations from an international panel of experts) include [12]: (1) a history of recurrent angioedema in the absence of concomitant hives or use of medication known to cause angioedema; (2) documented normal or near-normal C4, C1INH antigen, and C1INH function; and (3) either a genetic variant associated with the disease, or a strong family history of recurrent angioedema without hives, along with documented lack of efficacy of chronic high-dose antihistamine therapy, montelukast (unless contraindicated), and a course of omalizumab. Healthcare providers should have a strong index of suspicion for HAE-nC1INH if a patient presents with the above criteria and has not achieved benefit from corticosteroids and/or a trial of omalizumab. Testing for gene variants known to be associated with the condition (ie, *F12*, *ANGPT*, *PLG*, *KNG1*, *MYOF*, *HS3ST6*) should be performed where possible. The Guideline Authors affirmed that these diagnostic criteria are based on ideal feasibility and availability of the above tests and should not be considered absolute requirements to make the diagnosis of HAE-nC1INH.

Beyond these genomic biomarkers, there are promising biochemical biomarkers associated with the bradykinin-forming cascade (eg, plasma kallikrein, cleaved high-molecular-weight kininogen, serine proteases) and the endothelium (eg, vascular endothelial growth factor C, asymmetric dimethylarginine) that may become clinically useful in the future for evaluation of HAE-nC1INH [159, 160].

### Acute treatment of HAE with normal *C1INH*

#### Background

As discussed above, HAE-nC1INH is a rare disease that can be a challenge to diagnose with certainty. This creates a unique set of challenges for patients since treatments are hard to access without a diagnosis. Patients may also be stigmatized due to a lack of understanding of their condition, especially if they present regularly to the emergency department with angioedema attacks but have documented normal *C1INH*. It has been suggested, particularly for HAE-FXII and HAE-PLG, that bradykinin plays a role in the pathogenesis of HAE-nC1INH, leading to speculation that therapies used for HAE due to *C1INH* deficiency may also be beneficial in this group [161]. There is indirect evidence that antihistamine therapy is not effective in such patients [162, 163].

Recommendation 22 **pdC1INH is an effective therapy for the acute treatment of attacks in patients with HAE-nC1INH.**

**Level of Evidence: Low** (90.2% Agree, 4.9% Disagree, 4.9% Abstain)

**Strength of Recommendation: Strong** (90.7% Agree, 9.3% Disagree)

#### Clinical considerations

Although robust evidence from case series or controlled clinical trials is lacking for the treatment of attacks in HAE with normal *C1INH*, we recommend a therapeutic trial of agents that have shown benefit in individual cases, given the potential for severe or life-threatening presentations. The same recommendations for avoiding known triggers (eg, estrogen-containing oral contraceptives or replacement therapy, ACE inhibitors, DPP-IV inhibitors, neprilysin inhibitors) that apply to patients with HAE due to *C1INH* deficiency also apply to those with HAE-nC1INH [50–52].

Plasma-derived C1INH is an effective therapy for some patients and is recommended for the acute treatment of attacks in patients with HAE-nC1INH. The low level of evidence reflects the lack of evidence from randomized controlled trials in patients with HAE-nC1INH. Intravenous pdC1INH has shown efficacy in reducing the duration and intensity of angioedema attacks in some patients with HAE-nC1INH, in a mix of observational studies [164–167] as well as other more rigorously designed retrospective and prospective observational studies [22, 167–172]. This reported efficacy occurs even though the pathogenesis of the angioedema, by definition, is not caused by a deficiency in *C1INH* [19, 20, 169, 173].

Mechanistically, the rationale for using C1INH-directed therapy in patients with normal C1INH levels is not entirely clear. It has been proposed that exogenous pdC1INH may act by increasing the threshold for activation of the kallikrein–kinin system and thereby limiting bradykinin generation [174]. The heterogeneity of HAE-nC1INH genotypes may contribute to variability in therapeutic response, as some mutations could subtly influence interactions with C1INH or contact pathway regulation [12]. While the mechanistic rationale for bradykinin-targeted therapy may appear more direct (see **Recommendation 23**), clinical evidence supporting both C1INH- and bradykinin-targeted approaches in HAE-nC1INH remains limited, and treatment choice should be individualized based on the patient’s disease characteristics, prior response to treatment, and access/availability of treatment options.

Recommendation 23 **Icatibant is an effective therapy for the acute treatment of HAE attacks in patients with HAE nC1INH.**

**Level of Evidence: Low** (95.1% Agree, 2.4% Disagree, 2.4% Abstain)

**Strength of Recommendation: Strong** (90.9% Agree, 6.8% Disagree, 2.3% Abstain)

#### Clinical considerations

As with pdC1INH, there is low-level evidence suggesting that blocking bradykinin B2 receptors with icatibant is an effective intervention for treating angioedema attacks in some patients with HAE-nC1INH. Icatibant appears to be effective across a range of attack severities and anatomical locations, with most evidence accumulated in the more frequently reported causative variants (ie, HAE-FXII, HAE-PLG) [19, 125, 164, 165, 167, 169–172, 175–177].

The Committee affirmed that these recommendations generalize to all patients with HAE-nC1INH and are not subdivided by causative variant in this guideline.

Note that there are limited data on the use and efficacy of tranexamic acid and FP for acute treatment in HAE-nC1INH, and there is currently no available evidence for rhC1INH or sebetralstat in this patient population.

### Short-term prophylaxis

#### Background

STP refers to the practice of treating patients to reduce the risk of associated and consequent morbidity and mortality during a period when there may be an increased risk of having an angioedema attack.

It is well recognized that physical trauma, as can occur during medical, surgical, and dental procedures, can induce episodes of angioedema [178–180]. Upper airway manipulation, including during dental surgery and intubation poses a particularly high risk due to its association with upper airway swelling. However, even minor procedures can precipitate angioedema, and predictions of when this may occur cannot be made with certainty. Attacks can occur anywhere from hours to several days after a procedure [178]. Healthcare providers should educate their patients about the possibility of angioedema attacks happening within 48 h post-procedure, even if they have received STP [180–183]. Patients should have a strategy to access acute treatment in the event of a post-procedure attack.

It is also thought that other triggers, such as emotional stressors, can precipitate attacks. Individual patients may be aware of specific triggers that have been known to precipitate their attacks. Despite these observations, no controlled clinical trials have been conducted to assess the risk of specific procedures; the available data primarily come from personal experience, retrospective reviews, and surveys [178, 179, 182–184].

Recommendation 24 **Short-term prophylaxis should be considered prior to known patient-specific triggers and made available for any medical, surgical, or dental procedures in patients with HAE due to C1INH deficiency.**

**Level of Evidence: Low** (97.7% Agree, 2.3% Disagree)

**Strength of Recommendation: Strong** (97.7% Agree, 2.3% Disagree)

#### Clinical considerations

As described above, there are limited data on the specific risks associated with a broad range of medical, surgical, and dental procedures. One study assessed the risk of angioedema following surgery without pre-procedural prophylaxis and reported a range from 5 to 30%, regardless of procedure type or extent [178]. Dental extraction tends to carry a higher risk of triggering angioedema than dental cleaning or cavity restoration [181, 183], but it remains challenging to directly associate the risk of an attack with a specific procedure [178, 179]. While healthcare providers may choose to forego prophylaxis for low-risk procedures—particularly if similar procedures have not previously triggered attacks—the use of STP may still be appropriate. STP helps reduce the risk of procedure-related attacks, facilitates safe access to necessary care, and alleviates patient anxiety about the potential for an HAE attack [185].

Available data do not indicate how many patients have been denied, declined, or avoided necessary procedures due to perceived risks, or how many were not offered STP [185]. STP should be considered based on known patient-specific triggers and made available for all procedures.

This recommendation was intended to be broad in its scope, since the risk of appropriate STP would likely be minimal compared with any real or perceived risk of not using STP when determined necessary. Ultimately, the decision of whether to use STP should involve both the patient and their HAE healthcare provider.

Recommendation 25 **HAE-specific acute treatment should be available during and after any procedures in patients with HAE due to C1INH deficiency.**

**Level of Evidence: Consensus** (100% Agree)

**Strength of Recommendation: Strong** (96.7% Agree, 3.3% Disagree)

#### Clinical considerations

All patients should have two doses of appropriate on-demand therapy immediately available, as described in the section on **Acute treatment of HAE due to *C1INH* deficiency**. Even those who receive STP should have two on-demand treatments available, given the potential risk for breakthrough attacks [179].

Patients who receive STP should be observed postoperatively to monitor for potential HAE attacks. It is important for healthcare providers to establish a follow-up protocol, such as a phone call or another suitable method based on the centre’s practices, to ensure patient safety and address any concerns after the procedure.

Recommendation 26 **Intravenous pdC1INH or rhC1INH should be used for short-term prophylaxis in patients with HAE due to C1INH deficiency.**

**Level of Evidence: Low** (95.5% Agree, 4.5% Disagree)

**Strength of Recommendation: Strong** (97.6% Agree, 2.4% Disagree)

#### Clinical considerations

Product-approved indications vary by country/region (see Table 3). In Europe, Cinryze^®^ is licensed for use in adults at a dose of 1000 IU, administered within 24 h of an anticipated procedure [74], while Berinert^®^ 1000 IU is recommended within 6 h [70]. For pediatric patients, the European licensing specifies dosing by weight: 500 IU of Cinryze^®^ for children weighing 10–25 kg and 1000 IU of Cinryze^®^ for children weighing > 25 kg (administered within 24 h of an anticipated procedure) [74], or 15–30 IU/kg of Berinert^®^ (administered within 6 h) [70].

In one study, patients undergoing tooth extractions had a 30% risk of angioedema without prophylaxis, which decreased to 15% with 500 IU and 5% with 1000 IU of pre-procedural pdC1INH [179]. In another study, 17 patients (some possibly on LTP) received STP prior to a dental procedure and experienced no angioedema attacks. However, one patient who was not on LTP and declined STP developed an attack 48 h after a dental extraction, requiring emergency treatment [183]. Pre-procedural use of IV pdC1INH at a dose of 20 IU/kg reduced the incidence of angioedema attacks in children and adolescent patients with HAE and is recommended as close as possible to the start of high-risk procedures [186]. However, given that breakthrough attacks have occurred even with prophylactic pdC1INH at 1000 IU [179], at least two additional doses of on-demand treatment for attacks should be readily available for all patients with HAE.

A retrospective study of 51 patients diagnosed with HAE due to *C1INH* deficiency from the US and Europe evaluated the efficacy of rhC1INH administered as STP within several hours before high-risk procedures (primarily dental procedures and surgery) [184]. The median dose of rhC1INH was 3075 IU, administered a median of 60 min before the procedure. Without pre-procedural prophylaxis, 76.9% of procedures (22 of 26) resulted in angioedema attacks within 48 h. With STP with rhC1INH, 3.9% of procedures (2 of 51) resulted in angioedema attacks among patients not receiving concurrent LTP. Similar efficacy was observed in patients on LTP. These findings highlight the efficacy of rhC1INH in preventing attacks following medical procedures, supporting its use as STP for patients with HAE due to *C1INH* deficiency. Both rhC1INH and pdC1INH have demonstrated efficacy in preventing HAE attacks when administered pre-procedure, despite differences in half-lives. The key is for the patient and their HAE specialist to discuss appropriate dosing and timing prior to a procedure, while accounting for patient-specific considerations to minimize the risk of HAE attacks during and after the procedure.

Several patients reportedly used icatibant effectively as STP prior to dental procedures [187]. However, icatibant has a short half-life and should not be used as STP. Attenuated androgens or FP may be considered for STP when pdC1INH is not available, particularly if HAE-specific acute treatments are not available. When androgens are chosen for STP, danazol (2.5 to 10 mg/kg/day, up to a maximum 600 mg/day) can be considered, starting 5 days before the anticipated procedure or trigger and continuing 2–3 days after the anticipated trigger [31]. Disadvantages with androgen therapy include perceived inferior efficacy to pdC1INH concentrate. Attenuated androgens are not suitable in pregnancy or during breastfeeding, and a pregnancy test should be considered before initiation of therapy with androgens. Frequent short-term use may be associated with similar effects as long-term use of androgens as discussed in the section on **Long-term prophylaxis in HAE due to *C1INH* deficiency**. The optimal dose of FP for STP has not been determined, but based on cases in the literature, it is typically given as 2 U in adults and 10 mL/kg in children, 1 to 2 h prior to a procedure [94, 188–190].

Antifibrinolytic agents, such as tranexamic acid, have also been used for STP. The suggested dosage is 25 mg/kg administered two or three times daily, up to a maximum of 3–6 g per day, starting 5 days before and continuing for 2–5 days after a procedure. Antifibrinolytic agents should be used only if other therapies are not available [31].

Lanadelumab (Takhzyro^®^), is a humanized monoclonal antibody against kallikrein, that takes approximately 70 days to reach a steady-state concentration [191–193], and is therefore not recommended for STP. Similarly, SC pdC1INH requires approximately 3 weeks to reach a therapeutic steady-state [192], and berotralstat requires 6–12 days [193], which suggests that neither agent is appropriate for use as STP. Data are lacking as to whether STP is necessary if patients are symptom-free following initiation of LTP with berotralstat, donidalorsen, garadacimab, lanadelumab, or pdC1-INH (see **Recommendation 31**). Until such data are available, STP should be used, if indicated, when prescribing any effective treatment for LTP.

Recommendation 27 **Short-term prophylaxis is recommended in the case of a cesarean delivery or intra-partum instrumentation in patients with HAE due to C1INH deficiency. Effective acute therapy should be immediately available for all deliveries.**

**Level of Evidence: Consensus** (100% Agree)

**Strength of Recommendation: Strong** (89.7% Agree, 10.3% Disagree)

#### Clinical considerations

In general, pregnant patients with HAE do not appear to require routine STP for uncomplicated vaginal deliveries [110, 112, 113, 115]. However, there are certain instances in which STP could be considered prior to vaginal delivery, including in patients with a history of severe HAE attacks, frequent attacks during the third trimester, or edema secondary to mechanical trauma [111, 115, 194–196]. STP is recommended before a cesarean delivery or intrapartum instrumentation and may need to be repeated, subject to drug half-life, if delivery does not occur within a certain time frame [111, 113]. As with other procedures, two doses of on-demand therapy should be available in case of an HAE attack.

Recommendation 28 **Short-term prophylaxis should be considered prior to known patient-specific triggers and made available for any medical, dental, and surgical procedures for patients with HAE-nC1INH.**

**Level of Evidence: Consensus** (90.7% Agree, 9.3% Disagree)

**Strength of Recommendation: Strong** (86.4% Agree, 9.1% Disagree, 4.5% Abstain)

#### Clinical considerations

HAE-nC1INH is a rare condition, and due to the limited published data and lack of controlled clinical studies, there is little established guidance for its management. However, we believe it is necessary to make a recommendation for STP based on clinical experience and expert consensus, drawing on insights from the broader understanding of HAE pathophysiology. The recommendation to consider STP prior to known patient-specific triggers and to make STP available for any medical, dental, and surgical procedures for patients with HAE-nC1INH aims to optimize patient care, even in the absence of extensive evidence. This includes prior to delivery in pregnant patients with HAE-nC1INH [197]. As data become available, this recommendation may be refined, but it represents a best-practice approach in the current context.

### Long-term prophylaxis in HAE due to *C1INH* deficiency

#### Background

LTP refers to the use of ongoing, regular treatment to prevent attacks of HAE. LTP is indicated when on-demand treatment does not sufficiently meet patient treatment requirements, as discussed in the section on **Approach to individualized therapy**. Prophylactic therapy reduces the frequency, duration, and severity of attacks and ideally results in the patient becoming attack-free. The specifics of when to consider and when to initiate LTP are discussed below.

Recommendation 29 **Long-term prophylaxis should be considered at every visit taking disease activity, burden, control, and patient preference into account.**

**Level of Evidence: Consensus** (97.4% Agree, 2.6% Disagree)

**Strength of Recommendation: Strong** (100% Agree)

Recommendation 30 **All patients should have a management plan, including immediate access to effective treatment for attacks, even when on prophylaxis.**

**Level of Evidence: Low** (100% Agree)

**Strength of Recommendation: Strong** (100% Agree)

#### Clinical considerations

The goals of LTP are to: (1) reduce the frequency and/or severity of HAE attacks, ideally preventing attacks entirely; (2) minimize the impact of HAE on HRQoL; and (3) enable patients to lead normal lives. The benefits and risks of LTP should be explored through ongoing patient–physician discussions to optimize patient care (see section on **Shared decision-making**). No prophylactic regimen guarantees complete elimination of angioedema attacks. Therefore, patients on prophylaxis should be prepared to manage angioedema attacks as described in **Recommendation 3**, as part of a comprehensive acute treatment plan developed collaboratively between the patient and their physician. Considerations for initiating prophylaxis are outlined in the **Approach to individualized therapy** section. The decision to start LTP should be based on the therapy’s efficacy and safety, and the patient’s preferences, without cost being a determining factor.

Recommendation 31 **Berotralstat, donidalorsen, garadacimab, lanadelumab, and pdC1INH are effective therapies for long-term prophylaxis in patients with HAE due to C1INH deficiency.**

**Level of Evidence: High** (82.2% Agree, 17.8% Disagree)

**Strength of Recommendation: Strong** (93.2% Agree, 4.5% Disagree, 2.3% Abstain)

#### Clinical considerations

High-level evidence supports the use of various therapies for LTP in patients with HAE due to *C1INH* deficiency. The following section provides a summary of these therapies, with detailed data available in the systematic search findings (see Additional File 1 and Additional File 2). A brief overview of their efficacy and role in LTP is provided below, with approved indications and doses listed in Table 3. The following therapies are discussed in alphabetical order, without implying any hierarchy of preference.

Berotralstat (Orladeyo^®^) is a once-daily oral plasma kallikrein inhibitor that works by inhibiting plasma kallikrein activity to control excessive bradykinin generation, thus preventing bradykinin from causing HAE attacks [193]. It is approved in Canada, Europe, Japan, and the US for the routine prevention of HAE attacks in adults and pediatric patients aged 12 years and older. In some regions, approval includes a minimum body weight requirement of 40 kg [193, 198–201]. Berotralstat is typically administered as one 150-mg capsule once daily with food. In certain cases, such as patients with hepatic impairment, those receiving chronic treatment with P-glycoprotein or breast cancer resistance protein inhibitors (due to drug interactions), or those experiencing persistent gastrointestinal adverse effects, the dose may be reduced to 110 mg once daily. This lower dose is permitted in some regions, including Japan and the US [198, 199]. In the APeX-2 phase 3 trial (*N* = 120; adults and adolescents), berotralstat 150 mg daily significantly reduced HAE attack frequency compared with placebo (1.31 vs. 2.35 attacks/month, *P* < 0.001) [202]. More participants on berotralstat achieved ≥ 50% (58% vs. 25%, *P* = 0.005) and ≥ 70% (50% vs. 15%, *P* < 0.001) reduction in attacks, though ≥ 90% reduction in attacks (23% vs. 7.5%) showed no statistical difference (ad hoc analysis). Berotralstat reduced on-demand therapy use (1.29 vs. 2.79 doses/month, *P* < 0.001). HRQoL was measured using the Angioedema Quality of Life (AE-QoL) questionnaire, a 17-item, angioedema-specific questionnaire that allows for total score ranging from 0 to 100, with higher scores indicating greater impairment. Both the berotralstat 150 mg and placebo groups showed improvements, with mean changes (−14.59 points with berotralstat vs. −9.96 points with placebo) exceeding the minimal clinically important difference (a 6-point decrease) [202]. In the open-label period (Weeks 48–96), patients on berotralstat 150 mg had a 90.8% attack reduction from baseline, maintained clinically important AE-QoL improvements, and used less on-demand medication [203, 204]. The most frequently reported treatment-emergent adverse events were abdominal pain, vomiting, diarrhea, and back pain. Gastrointestinal adverse events were mild and self-limited [202–204]. Similar findings were seen in the APeX-J trial in Japan (*N* = 19) [205, 206].

Donidalorsen, which inhibits plasma prekallikrein by targeting its RNA, has been studied in phase 2 (*N* = 20 adults) and phase 3 (*N* = 90 adults and adolescents) trials [207, 208]. By Week 17 of the phase 2 study, four doses of SC donidalorsen 80 mg every 4 weeks reduced angioedema attacks by 90% (*P* < 0.001) and improved AE-QoL scores (–26.8 vs. –6.2 points, *P* < 0.001), compared with placebo [207]. By Week 25 of the phase 3 study, greater reductions in attack rate were observed with more frequent dosing: 81% reduction (every 4 weeks) and 55% reduction (every 8 weeks) versus placebo [208]. The 4-week group also showed significant reductions in moderate/severe attacks (89%) and use of on-demand therapy (92%) compared with the placebo group (*P* < 0.001). Moreover, 53% of patients remained attack-free (vs. 9% in the placebo group; *P* = 0.003). Numerically greater proportions of participants achieved ≥ 50% reduction in attacks with 4-weeks (93%) and 8-week donidalorsen dosing (83%) compared with the placebo group (27%). Statistically significant greater proportions achieved ≥ 70% reduction in attacks (82% and 65% vs. 18%, *P* < 0.001 and *P* = 0.004, respectively). Numerically greater proportions achieved ≥ 90% reduction in attacks (62% and 48% vs. 9%), but this endpoint did not reach statistical significance. AE-QoL improved by 24.8 points with 4-week dosing (18.6-point treatment difference vs. placebo, *P* < 0.001), and 91% achieved well-controlled disease (Angioedema Control Test [AECT] ≥ 10 points) by Week 25, compared with 41% in the placebo group [208]. Common adverse events included injection-site erythema and headache [208]. Although donidalorsen had not received marketing authorization for HAE due to *C1INH* deficiency at the time the recommendations were developed, it was included in the **Recommendation 31** based on high-quality evidence supporting its use. Subsequently, donidalorsen received regulatory approval in the US in August 2025 [209].

Garadacimab (Andembry^®^) is a monoclonal antibody that inhibits activated FXII. Garadacimab was evaluated in a 6-month phase 3 trial (*N* = 64 adults and adolescents) as a 200-mg SC injection administered monthly, starting with a 400-mg loading dose [210]. It reduced the mean attack rate by 87% compared with placebo (0.27 vs. 2.01 attacks/month, *P* < 0.0001). In the first 3 months, 72% of participants in the garadacimab group had no attacks compared with 8% in the placebo group. A reduction in attack frequency ≥ 90% was achieved by 74% versus 8% (*P* < 0.0001). Garadacimab also reduced moderate/severe attacks by 90% (0.13 vs. 1.35 attacks/month, *P* < 0.0001) and reduced attacks requiring on-demand therapy by 88% (0.23 vs. 1.86 attacks/month, *P* < 0.0001). The safety profile was similar to placebo; common adverse events included upper respiratory tract infection, headache, and injection-site reactions [210]. Although garadacimab had not received marketing authorization for HAE due to *C1INH* deficiency at the time of the Conference, it was included in the **Recommendation 31** based on high-quality evidence supporting its use. Since the Conference, approvals have been granted in Australia, Canada, Europe, Japan, and the US for use in adults and adolescents ≥ 12 years [211–216].

Lanadelumab is a fully humanized, SC injectable monoclonal antibody (immunoglobulin G1 with kappa light chains) that targets active plasma kallikrein. It is approved in Canada and Japan for adults and adolescents ≥ 12 years [191, 217], and in Australia, Europe, and the US for broader use in patients ≥ 2 years [218–221]. For adults and adolescents, it is typically administered as 300 mg every 2 weeks; but for well-controlled patients (eg, attack-free for more than 6 months), a dosing interval of 300 mg every 4 weeks may be considered [191, 218, 220]. In the HELP phase 3 trial (*N* = 125, ≥ 12 years), lanadelumab significantly reduced the frequency and severity of HAE attacks compared with placebo. After 6 months, attack rates were reduced by 83.1% (150 mg every 4 weeks), 96.6% (300 mg every 4 weeks), and 97.2% (300 mg every 2 weeks) versus 20.8% with placebo (*P* ≤ 0.001). Improvements were observed as early as 2 weeks [222]. Additionally, lanadelumab significantly improved AE-QoL scores, compared with placebo (–19.5 vs. –4.7 points; *P* < 0.01). Notably, 70% of participants on lanadelumab achieved the minimal clinically important difference (6-point reduction) in AE-QoL scores, compared with 37% who received placebo [223]. Injection-site pain, headache, viral upper respiratory tract infection, and injection-site erythema were the most commonly occurring treatment-emergent adverse events with lanadelumab treatment [222].

Phase 3 open-label studies, open-label extensions (OLE), and real-world analyses reinforce the phase 3 trial results, confirming lanadelumab as an effective LTP treatment option [149, 224–226]. In the HELP OLE, lanadelumab reduced HAE attack rates by 87.4% from baseline, with 37.3% of participants remaining attack-free throughout the study (81.6% completed ≥ 30 months) [224]. Real-world analyses demonstrated a significant reduction in attack frequency, from 6.4 to 0.3 attacks per month, and improvements in AE-QoL [226].

Prophylaxis with pdC1INH is available as IV or SC formulations, typically administered every 3–4 days, with specific dosing and age indications varying by region (refer to Table 3). Controlled clinical trials have demonstrated that both IV and SC pdC1INH therapies are effective for LTP in patients with HAE due to *C1INH* deficiency, significantly reducing the number, duration, and severity of attacks [227–229]. In a randomized controlled trial, IV pdC1INH reduced the mean number of attacks normalized over 12 weeks from 12.73 to 6.26, corresponding to a mean difference of 6.47 attacks (*P* < 0.001) [227]. In the phase 3 COMPACT trial, SC pdC1INH reduced monthly attack rates compared with placebo by 2.42 attacks (40 IU/kg) and 3.51 (60 IU/kg), both statistically significant (*P* < 0.001) [228]. Recent clinical evidence has confirmed that SC administration is highly efficacious, [230–234] offering a convenient alternative to IV dosing. In a post hoc analysis of the phase 3 COMPACT trial, SC pdC1INH prophylaxis demonstrated a preventive treatment effect, with evidence of benefit within 2 weeks. Fewer participants on 40 IU/kg (28%) and 60 IU/kg (23%) experienced attacks, compared with placebo (89% and 81%, respectively) [234]. In the COMPACT OLE study, treatment with 60 IU/kg significantly reduced attack frequency, with nearly half of participants experiencing fewer than one attack per year [230, 231]. The most frequently reported adverse events were nasopharyngitis, upper respiratory tract infections, headache, and localized injection-site reactions [231]. Additionally, improvements in AE-QoL were observed, particularly in work productivity and anxiety [233]. The SC route also reduces the inconvenience and medicalization associated with IV administration, and avoids hazards of repeated venipuncture and indwelling catheters [235], further improving HRQoL [236].

Although donidalorsen and garadacimab had not yet received marketing authorization for HAE due to *C1INH* deficiency at the time of the Conference, both treatments were included in the recommendations based on high-quality evidence supporting their use. Since the Conference, approvals have been granted in several regions (see Table 3), further reinforcing their anticipated role in clinical practice.

As a result of this high-quality body of evidence, the Guideline Authors recommended berotralstat, donidalorsen, garadacimab, lanadelumab, and pdC1INH as effective LTP treatments for patients with HAE due to *C1INH* deficiency.

#### Recommendation 32

**Berotralstat, donidalorsen, garadacimab, lanadelumab, or pdC1INH could be used as first-line therapy for long-term prophylaxis in patients with HAE due to C1INH deficiency.**

**Level of Evidence: Consensus** (100% Agree)

**Strength of Recommendation: Strong** (95.5% Agree, 4.5% Disagree)

#### Clinical considerations

Given the high-quality evidence for berotralstat, donidalorsen, garadacimab, lanadelumab, and pdC1INH, and in the absence of head-to-head comparisons, we strongly agree that any of these agents can be considered suitable first-line LTP treatment options. Patients and their HAE specialists should collaborate closely to select the most appropriate treatment, recognizing that not all patients respond equally to every treatment, and that having multiple options available is important.

While androgens and antifibrinolytics are no longer recommended as first-line options for LTP, they may be considered for patients who have previously benefitted from their use or who face barriers to accessing first-line therapies. It should not be necessary for patients to try androgens or antifibrinolytics before considering first-line treatments, such as berotralstat, donidalorsen, garadacimab, lanadelumab, or pdC1INH.

Androgens, such as danazol, have been shown to reduce the frequency and severity of HAE attacks [237–242], but evidence is limited, due to study quality concerns [240]. Historically, many patients have been controlled with androgen therapy, and its use in some patients may be acceptable provided that the lowest effective dose (≤ 200 mg/day) is used to achieve efficacy and minimize adverse events [161, 243]. However, androgens are associated with many potential adverse effects. These agents can affect serum lipid levels, cause hepatotoxicity or hepatitis, and have been associated with hepatocellular adenoma and, in very rare cases, carcinoma [238, 244, 245]. It is recommended that all patients on androgen therapy be monitored for hypertension and have a complete blood count, liver enzymes, urinalysis, serum α-fetoprotein, creatine phosphokinase, and lipid profile every 6 months, as well as an annual liver ultrasound [14].

Virilizing effects of androgen therapy can occur, including menstrual irregularities, masculinization, irreversible voice alteration, and hirsutism. Psychological side effects include emotional irritability and lability, aggressive behaviour, depression, and anxiety disorders. Androgens interact with several medications. They are contraindicated in pregnancy and lactation, before puberty, and in patients with androgen-dependent malignancy or hepatitis [244, 245]. Patients need to be made aware of these side effects when considering androgen therapy, and while on it, and physicians should carefully consider the individual patient’s risk profile.

Tranexamic acid, an antifibrinolytic, has moderate evidence supporting its use in LTP for HAE [246, 247]. However, due to its limited efficacy and potential side effects, it is currently considered less relevant for most patients. The recommended dosage is 30–50 mg/kg daily, divided into two or three doses, up to a maximum of 6 g per day.

A phase 2, randomized, placebo-controlled crossover trial has demonstrated that prophylaxis with rhC1INH can reduce the frequency of HAE attacks [248]. Where other first-line therapies are unavailable, HAE specialists may consider the published data on rhC1INH, though its use in LTP is not approved.

### Long-term prophylaxis in pregnant, breastfeeding, or lactating patients with HAE

#### Background

When selecting LTP for pregnant patients, healthcare providers should carefully evaluate the efficacy and safety of treatment for both mother and infant during pregnancy, labour and delivery, and throughout the breastfeeding/lactation period. Given the rarity of HAE combined with the ethics of enrolling pregnant patients in clinical trials, it is unlikely there will be any placebo-controlled interventional studies assessing LTP treatment options prospectively in pregnant patients with HAE. The available evidence comes from open-label trials, observational studies, case reports, retrospective reviews, questionnaires, and expert opinion.

As mentioned in the section on **Acute treatment in pregnant, breastfeeding, or lactating patients with HAE**, angioedema attacks may persist during and after the breastfeeding/lactation period, although at lower rates than during pregnancy [114]. Therefore, the Guideline Authors agreed there is a need to specifically address breastfeeding/lactation in patients with HAE on LTP.

Recommendation 33 **Long-term prophylaxis should be considered in pregnant patients with HAE.**

**Level of Evidence: Consensus** (100% Agree)

**Strength of Recommendation: Strong** (97.4% Agree, 2.6% Disagree)

#### Clinical considerations

Pregnant patients with HAE may experience more frequent and severe attacks due to the hormonal changes of pregnancy, and are therefore more likely to opt for LTP to reduce disease burden and improve their HRQoL [249]. LTP is particularly important for patients experiencing severe or frequent attacks, or when attacks contribute to anxiety, depression, post-traumatic stress, or reduced HRQoL [249]. Although there are no clinical studies on the safety and efficacy of LTP treatments during pregnancy, multiple expert consensus panels have discussed treatment options and published recommendations for management during this period [14, 15].

Supporting both the physical and mental health of pregnant patients with HAE is essential, as pregnancy is inherently stressful and the added burden of HAE attacks can further impact well-being. Recurrent abdominal pain may be mistaken for labour contractions or primary gastrointestinal disease, leading to inappropriate clinical decisions. The growing uterus and fetus can trigger an angioedema attack, further complicating the pregnancy [250]. LTP should be discussed at every visit with pregnant patients with HAE with shared decision-making guiding the need for its use (see section on **Shared decision-making**).

Recommendation 34 **pdC1INH is the treatment of choice for long-term prophylaxis in pregnant patients with HAE.**

**Level of Evidence: Low** (100% Agree)

**Strength of Recommendation: Strong** (100% Agree)

#### Clinical considerations

In nonpregnant individuals with HAE, the use of pdC1INH for LTP of HAE attacks is well established [31, 227, 231]. Data from open-label trials [231, 251], observational studies [114, 252], and retrospective reviews [112, 113] demonstrated that pdC1INH was generally safe and not associated with any treatment-related adverse events during the study periods or neonatal abnormalities in those who became pregnant during treatment and subsequently discontinued treatment (mandated by trial protocols). Although the data in pregnant patients with HAE were not of high quality, we strongly recommended pdC1INH when LTP is required in pregnancy.

The most common adverse event is headache for IV pdC1INH [140] and local injection-site reaction for SC pdC1INH [253]. There is a rare risk of anaphylaxis [126]. pdC1INH products are currently manufactured to meet strict quality control standards. Despite these precautions, pdC1INH products are derived from human blood and there remains a theoretical risk that pasteurization and nanofiltration steps may not fully inactivate and eliminate all known and unknown blood-borne infectious agents. A special warning for thrombosis exists [74, 253]; however, many reported cases arose from the use of indwelling catheters [235, 254], which is now discouraged, or to administration of excessively high doses [255].

Recommendation 35 **Attenuated androgens should not be used in patients with HAE during pregnancy.**

**Level of Evidence: Consensus** (100% Agree)

**Strength of Recommendation: Strong** (93.2% Agree, 6.8% Disagree)

#### Clinical considerations

Androgens are contraindicated during pregnancy since these drugs can have significant effects on the normal development of the fetus, including masculinization. Potential effects on the female fetus include clitoral hypertrophy, labial fusion, urogenital sinus defect, vaginal atresia, and ambiguous genitalia [111, 256–258].

Antifibrinolytics may be considered when pdC1INH concentrate is unavailable. Although the efficacy and safety of antifibrinolytics as LTP is debatable in individuals with HAE due to *C1INH* deficiency [15], these agents may have greater potential in individuals with HAE-nC1INH [164, 259].

Limited clinical trial data suggest prophylaxis with rhC1INH may reduce HAE attack frequency, though it is not approved for LTP. While evidence on its safety during pregnancy is limited [80, 81]; there no evidence of harm and no theoretical reason to expect it. This may be relevant in situations where rhC1INH is the only available option, such as during shortages of pdC1INH.

Although berotralstat [193, 198, 200] and lanadelumab [218] are approved for LTP, there is insufficient evidence on the safety of these agents when used as LTP in pregnant patients with HAE. Lanadelumab is an immunoglobulin G1 monoclonal antibody that crosses the placental barrier [191, 218, 220], predominantly during the third trimester of pregnancy [126, 218]. The potential effects on the fetus are therefore likely to be greater toward the end of the pregnancy when the fetus is well formed. However, there is no evidence to suggest safe use of lanadelumab during pregnancy. As a precaution, lanadelumab should be stopped several weeks before attempting conception (due to its long half-life of approximately 2 weeks [260]). There are no data from the use of berotralstat in pregnant women; consequently, berotralstat is not recommended during pregnancy and should be avoided for at least five half-lives (ie, 20 days based on a median elimination half-life of 93 h) before attempting conception [198, 200].

Recommendation 36 **Long-term prophylaxis should be considered in patients with HAE during the breastfeeding/lactation period.**

**Level of Evidence: Consensus** (95.0% Agree, 2.5% Disagree, 2.5% Abstain)

**Strength of Recommendation: Strong** (97.4% Agree, 2.6% Abstain)

#### Clinical considerations

We strongly recommended that LTP should be considered in patients with HAE during the breastfeeding/lactation period given that angioedema attacks may be triggered by hormonal changes that occur during breastfeeding/lactation [111, 114, 116]. LTP should be discussed at every visit with breastfeeding/lactating patients with HAE, with shared decision-making guiding the determination for its use (see section on **Shared decision-making**).

Recommendation 37 **pdC1INH is the treatment of choice for long-term prophylaxis in breastfeeding/lactating patients with HAE.**

**Level of Evidence: Consensus** (100% Agree)

**Strength of Recommendation: Strong** (93.2% Agree, 6.8% Disagree)

#### Clinical considerations

Despite the lack of well-controlled studies on this topic, pdC1INH appears to be safe and effective as LTP during breastfeeding/lactation [111]. pdC1INH has been used in patients with HAE in Europe for more than 40 years and has demonstrated a favourable safety profile in pregnancy and breastfeeding/lactation, with no alternative treatment currently available for these individuals. Therefore, we strongly recommended pdC1INH as the treatment of choice for LTP during breastfeeding/lactation.

Recommendation 38 **Attenuated androgens should not be used in patients with HAE during the breastfeeding/lactating period.**

**Level of Evidence: Consensus** (100% Agree)

**Strength of Recommendation: Strong** (97.7% Agree, 2.3% Disagree)

#### Clinical considerations

Androgens are contraindicated during breastfeeding/lactation as they are secreted into human milk and cause masculinization and other adverse events in breastfed infants [111, 257, 258].

There are no data regarding the use of lanadelumab [193, 198, 200] or berotralstat [193, 198, 200] during lactation. It is unknown whether either agent is excreted in human milk. Many drugs can be excreted in human milk, and since a risk to the breastfed infant cannot be excluded, precaution should be exercised. The decision to resume lanadelumab or berotralstat should involve balancing the clinical need for treatment and potential harms against the benefits of breastfeeding/lactation.

### Long-term prophylaxis in pediatric patients with HAE due to *C1INH* deficiency

#### Background

Although HAE attacks are rare in patients under the age of 6 years, the frequency and severity of symptoms may increase over time, requiring adjustments in treatment to address the patient’s evolving needs [261–263]. LTP in the pediatric population needs to be flexible to accommodate changes in a patient’s hormones, stressors, and lifestyle [131].

Data from numerous clinical trials with novel medicines have been published since the previous edition of this guideline [149, 202–206, 208, 210, 222–225, 230–234, 264, 265], though only two trials focused on children [266, 267]. In trials involving both adolescents and adults, the adolescent population was small, and many studies did not analyze this age group separately. Consequently, the experiences gained from clinical practice will provide further insights into the long-term safety of prophylaxis regimens in pediatric patients.

Recommendation 39 **When long-term prophylaxis is indicated in neonates and infants (aged 0–2 years), pdC1INH is the treatment of choice.**

**Level of Evidence: Consensus** (93.4% Agree, 2.6% Disagree)

**Strength of Recommendation: Strong** (97.6% Agree, 2.4% Disagree)

#### Clinical considerations

Considering disease activity, burden, control, and patient/family preference in line with **Recommendation 29** apply equally to the pediatric population. The goal remains to reduce the frequency and severity of HAE attacks, minimize disease impact on HRQoL, and enable patients in this age group to live normal lives. This recommendation is informed by the collective clinical experience of the Guideline Authors, as no clinical trials have been conducted to date in children with HAE younger than 2 years of age. In the absence of direct evidence, expert opinion and extrapolation from older pediatric populations support the use of pdC1INH as the treatment of choice for this age group.

Recommendation 40 **When long-term prophylaxis is indicated in children and adolescents, lanadelumab and pdC1INH are effective treatments.**

**Level of Evidence: Moderate** (97.7% Agree, 2.3% Abstain)

**Strength of Recommendation: Strong** (100% Agree)

#### Clinical considerations

Three treatments are indicated for LTP in children (aged 2 to < 12 years) and/or adolescents (aged 12–17 years) with HAE due to *C1INH* deficiency. These treatments are lanadelumab, pdC1INH, and berotralstat. All are authorized for self-administration after appropriate training (see Table 3 for approved indications).

Lanadelumab, a SC injectable monoclonal antibody that targets active plasma kallikrein, is indicated for routine prevention of recurrent HAE attacks in patients aged ≥ 12 years in Canada and Japan [191, 217] and ≥ 2 years in the US and Europe [218, 220]. Refer to Table 3 for approved indications in other regions. Evidence supporting the use of lanadelumab is derived from the phase 3, open-label SPRING trial in pediatric patients aged 2–11 years [267] and the phase 3 randomized controlled HELP trial and its OLE in adolescent patients aged ≥ 12 years [224, 268, 269].

In the SPRING trial, lanadelumab 150 mg every 2 or 4 weeks for 52 weeks reduced monthly attack rates by 94.8%, from 1.84 attacks at baseline to 0.08 attacks at Week 52. Reductions were also seen in attacks requiring acute therapy (93.4%), moderate or severe attacks (96.8%), and high-morbidity attacks (95.2%). Overall, 76.2% of participants remained attack-free. Clinically meaningful improvements in HRQoL were observed across pediatric subgroups, with Pediatric Quality of Life Inventory Total Score improvements of + 30.63 points (ages 2–4 years), + 30.00 points (5–7 years), and + 14.31 points (8–11 years), all exceeding the 4.5-point threshold. Adverse events were generally mild, with injection-site pain being the most common [267].

In the HELP OLE, lanadelumab 300 mg every 2 weeks reduced monthly attack rates by 94.7% in adolescent patients (*N* = 21) from 1.58 at baseline to 0.11 at study end, with 95.2% completing ≥ 30 months. AE-QoL scores improved from 27.5 to 7.5 points, surpassing the minimum clinically important difference (6-point reduction). While 57.1% of participants reported treatment-related adverse events, none were serious [269].

Intravenous pdC1INH (Cinryze^®^) is indicated for the routine prophylaxis of angioedema attacks in patients with HAE aged ≥ 6 years in the US and Europe [74, 140], and for those aged ≥ 12 years in Canada [141]. A phase 3, randomized, single-blind, crossover study assessed the efficacy of twice-weekly 500 IU or 1000 IU IV pdC1INH in 12 children aged 6–11 years. The study found a 76.2% reduction in the monthly normalized number of attacks with 500 IU and 87.4% reduction with 1000 IU pdC1INH. The reduction with 1000 IU was significantly greater than with 500 IU (*P* = 0.035). Additionally, the number of attacks requiring acute treatment decreased. No serious treatment-emergent adverse events were reported [266].

Subcutaneous pdC1INH (Haegarda^®^, marketed under various names internationally: Berinert^®^ SC in Australia, Berinert^®^ 2000/3000 in Europe, and Berinert^®^ S.C. Injection in Japan) is indicated for the routine prevention of angioedema attacks in patients with HAE. The approved age varies by region: no age restriction in Japan [270], ≥ 6 years in the US [253], ≥ 8 years in Australia [271], and ≥ 12 years in Canada and Europe [192, 272–274]. A pediatric subanalysis of the COMPACT OLE evaluated the long-term safety and efficacy of SC pdC1INH (40 or 60 IU/kg twice-weekly) in 10 patients aged 6–17 years over 52 weeks (104 weeks in the US) [265]. All participants showed ≥ 50% reduction in attacks compared with baseline and had < 1 attack per 4-week period. The median reduction in attacks was 97%, with a monthly attack rate similar to adults (0.11 vs. 0.09 attacks/month). Among 38 attacks, 42% were treated with acute therapy. Injection-site erythema was reported by two participants. There were no serious adverse events [265].

We concluded that these data provided a moderate level of evidence. We strongly recommend that, when LTP is indicated for children and adolescents, lanadelumab and pdC1INH are both effective treatment options. Although berotralstat is approved for patients aged ≥ 12 years in Japan and the US, and for those aged ≥ 12 years and weighing ≥ 40 kg in Canada and Europe, there are insufficient data in children younger than 12 years to support its inclusion in **Recommendation 40**.

Recommendation 41 **Berotralstat is an effective therapy for long-term prophylaxis in adolescent patients with HAE due to C1INH deficiency.**

**Level of Evidence: Low** (100% Agree)

**Strength of Recommendation: Strong** (86.1% Agree, 13.9% Disagree)

#### Clinical considerations

Several studies investigating the efficacy and safety of berotralstat have included a small number of pediatric patients, although not all provided specific analyses for this subgroup [202, 205, 206]. The global, open-label APeX-S study evaluated the long-term efficacy and safety of daily oral berotralstat (110 or 150 mg) in patients with HAE, including both adolescents (aged 12–17 years) and adults (aged ≥ 18 years) [275]. In the adolescent subgroup, the mean (standard deviation) attack rates over the 48-week treatment period were 0.77 (0.96) attacks/month for the 150-mg dose group (*n* = 5) and 0.78 (1.02) attacks/month for the 110-mg dose group (*n* = 5). Three of the participants experienced treatment-related adverse events, including abdominal pain, headache, and dizziness, and one patient in the 110-mg group discontinued treatment due to Grade 2 upper abdominal pain [275].

Despite the limited pediatric data, which resulted in a lower level of evidence, we strongly recommended berotralstat as an effective LTP therapy for adolescent patients with HAE due to *C1INH* deficiency.

Although adolescent participants were included in the clinical trials of donidalorsen [208] and garadacimab [210], the number enrolled did not meet the predefined threshold for inclusion in the Conference discussions (ie, at least five subjects per treatment arm). As such, these agents were not discussed for use in the adolescent population and were not included in **Recommendation 41**.

Recommendation 42 **Androgens should not be used for long-term prophylaxis in paediatric patients.**

**Level of Evidence: Moderate** (100% Agree)

**Strength of Recommendation: Strong** (92.7% Agree, 7.3% Disagree)

#### Clinical considerations

Androgens are known to cause premature epiphyseal closure [276, 277], among other significant side effects, making them contraindicated as LTP in the pediatric population before Tanner Stage 5. However, due to their efficacy and in the absence of other available options, androgens may be considered once patients have completed puberty. If androgen use is necessary, pediatric patients should start at the lowest effective dose and undergo regular monitoring for potential side effects [131].

Antifibrinolytics cannot be recommended for LTP in the pediatric population due to a lack of evidence. When they have been studied in children, their efficacy has been limited [131].

Similar to adults, pediatric patients should not be required to trial nonspecific therapies, such as androgens or antifibrinolytics, before proceeding to more specific LTP agents.

### Long-term prophylaxis treatment in HAE with normal *C1INH*

#### Background

Patients with HAE-nC1INH share similar clinical characteristics as patients with HAE due to *C1INH* deficiency, including the risk of random, unpredictable attacks of debilitating and potentially life-threatening angioedema [162]. These similarities have led to speculation that treatments used for LTP for HAE due to *C1INH* deficiency may also be beneficial for HAE-nC1INH. There is increasing anecdotal evidence supporting the use of LTP treatment in patients with HAE-nC1INH, which has led the Guideline Authors to include a recommendation for its use.

Recommendation 43 **Long-term prophylaxis may be appropriate for some patients with HAE-nC1INH. The decision to start a patient on long-term prophylaxis should be individualized between an HAE specialist and the patient.**

**Level of Evidence: Low** (90.2% Agree, 7.3% Disagree, 2.4% Abstain)

**Strength of Recommendation: Strong** (97.7% Agree, 2.2% Disagree)

#### Clinical considerations

Favourable responses to LTP with various agents have been reported in the literature, primarily through case reports, case series, and retrospective studies. Although responses to specific treatments may vary by HAE-nC1INH variant, the lack of clinical trial data makes it difficult to draw definitive conclusions regarding variant-specific efficacy.

A survey of US physicians revealed that 54% of their patients with HAE-nC1INH receive LTP, with the primary reasons for initiating treatment being high attack frequency and severity. Patients who started LTP experienced a reduction in attacks, from an average of 16 per year prior to initiating LTP to eight attacks per year after. Physicians typically recommended LTP for those with 10 or more attacks annually, with lanadelumab and SC pdC1INH being the most prescribed therapies [11].

Positive responses have been observed in patients with HAE-nC1INH using anabolic androgen, berotralstat, lanadelumab, pdC1INH, progestin, rhC1INH, or tranexamic acid to prevent HAE attacks [12, 123, 163, 278, 279]. Patients with specific variant subtypes, such as HAE-FXII [23, 24, 165, 167, 169, 170, 280, 281], HAE-PLG [172, 175, 281–283], and HAE-nC1INH with unknown mutations [170, 280, 281] have shown favourable responses to LTP, particularly with tranexamic acid. Favourable responses to lanadelumab [284] and progestin [23, 24, 169] have been documented in HAE-FXII; however, there is limited evidence for other HAE-nC1INH variants.

This body of low-level evidence suggests the potential effectiveness of LTP in managing HAE-nC1INH, supporting the recommendation that LTP may be appropriate for some patients. The decision to initiate LTP should be individualized, with input from both the patient and an HAE specialist, as described in the sections on **Approach to individualized therapy** and **Shared decision-making**.

Effective prevention of HAE attacks in patients with HAE-nC1INH requires careful treatment selection based on disease pathophysiology, genetic factors, and a structured process to identify the most effective therapies. Mast cell–mediated angioedema must be ruled out. Genetic testing plays a crucial role in guiding treatment decisions, though it may not be available in all centres. Patients should ideally be managed by specialists with expertise in HAE. Additionally, it is important to avoid polypharmacy, document treatment efficacy and tolerability, and be cautious of over-treatment. Randomized controlled trials enrolling patients with defined genetic mutations are needed to refine treatment strategies and improve outcomes. Ongoing efforts to identify an efficacious bradykinin-targeting on-demand therapy will be essential to managing patients with HAE-nC1INH.

Finally, we continue to emphasize the importance of avoiding known triggers of angioedema, such as estrogen-containing oral contraceptives or replacement therapy, ACE inhibitors, DPP-IV inhibitors, and neprilysin inhibitors [50–52].

### Self-administration

#### Background

Self-administration refers to the treatment of patients outside of a healthcare facility, either by the patients themselves or by a trained caregiver. The recognition and support of self-administration of treatment for HAE date back to the first international consensus document on HAE in 2003, and have been repeatedly affirmed in subsequent consensus statements and guidelines [14, 243, 285]. Self-administration has been shown to be a safe and convenient option for patients, enabling early treatment, and potentially lowering overall treatment costs compared with hospital-based therapy for HAE [286, 287]. Responses to two online surveys distributed to physicians in the US revealed a significant trend toward patient empowerment via self-administration, with the home (rather than the emergency department) being the predominant treatment setting for HAE attacks [288]; 54% of HAE attacks were treated at home in 2019 compared with 12% in 2010 and 33% in 2013. Furthermore, 90% of physicians surveyed and 84% of their patients with HAE expressed a preference for self-administration [289]. Among physicians, 67% reported that SC injections were the most commonly prescribed route for prophylactic treatment; 54% of patients indicated satisfaction with this method [289]. Although specific data in Canada are lacking, there is little reason to believe that findings would differ significantly in this country.

Treatment is more efficacious when HAE attacks are treated early [290, 291]. Evidence indicates that treating an attack early leads to faster symptom resolution and shorter attack duration [64, 65, 291]. Timely treatment of an attack relies on minimizing the steps between recognizing the need for and starting effective therapy. Requiring patients to travel to a healthcare facility for a therapy proven effective when administered at home or in nonclinical settings can lead to treatment delays [292], and may result in many attacks going untreated. Patients may also face difficulties accessing treatment if local healthcare facilities are unfamiliar with HAE. All therapies should be made available worldwide to all patients with HAE, and home-administration and self-administration are preferred because they reduce morbidity, absenteeism, cost, disease burden, and potentially mortality, as well as improve HRQoL [14, 293–295].

Recommendation 44 **All patients with HAE should be trained on self-administration of HAE-specific therapies if they are suitable candidates. If patients cannot self-administer therapy, provisions should be made to ensure timely access to all appropriate therapies.**

**Level of Evidence: Consensus** (100% Agree)

**Strength of Recommendation: Strong** (100% Agree)

#### Clinical considerations

Although the level of evidence was revised to consensus, this reflects the current lack of robust data rather than a change in the strength of the recommendation that all patients should be trained on self-administration of HAE-specific therapies if they are suitable candidates. The unanimous agreement among experts highlights the extensive clinical experience supporting self-administration as a critical element of HAE management. This recommendation is consistent with prior consensus statements and guidelines [14, 15, 31]. The importance of early therapy should not be underestimated, and barriers that affect its implementation should be removed. Geographic and regional disparities in care are known to exist, and self-administration of therapy helps to mitigate these issues [14, 296]. Although pdC1INH requires special considerations, including product tracking and patient training, the use of blood products for self-administration is not unique to HAE. Self-administration has a long-established history in hemophilia care and has been shown to be effective [297, 298]. Another example is self-administration of SC immunoglobulin for patients with primary immunodeficiency disease [299, 300]. Additionally, self-management has long been an integral part of care for chronic conditions, such as diabetes mellitus, and structured plans have also been developed to help individuals at risk of anaphylaxis recognize early symptoms and initiate timely treatment [301].

Several characteristics of HAE make it particularly well-suited for self-treatment via SC, IV, and especially oral administration. As a rare, potentially life-threatening condition with unpredictable attacks, early intervention is critical, and prompt treatment can significantly improve outcomes. LTP is often required, and both SC/IV administration and oral therapy offer effective and convenient methods for ongoing management. STP may also be required prior to anticipated exposure to various triggers. Self-treatment empowers patients to become experts in their own care, helping them to maintain independence and control, which can lead to improvements in their QoL [301]. The availability of SC-administered HAE therapies has further enhanced the ease of self-administration (see Table 3) [288, 289, 293]. However, injectable therapies can pose barriers to some patients due to factors such as needle phobia, poor venous access, or impaired dexterity, which can make SC/IV self-administration difficult. For patients who cannot self-administer injectable treatments, oral therapy (ie, berotralstat) provides a viable alternative for LTP.

Although not all patients will be suitable candidates for self-administered therapy, the vast majority are, and this option should be incorporated in the comprehensive care plan for patients with HAE. Patients deemed appropriate candidates for self-administration and willing to learn should adhere to recommendations outlined in established international home-therapy consensus statements [302, 303]. If patients are unable to self-administer therapy, provisions should be made to ensure timely access to all appropriate treatments, including through the training of caregivers to administer therapy when necessary [31]. With self-administered therapy, patients need to be regularly monitored to ensure appropriate control of their symptoms, compliance, and competency. This is discussed further in the **Approach to individualized therapy** section below.

### Approach to individualized therapy

#### Background

HAE is a dynamic chronic disease, and attacks of angioedema can vary in frequency and severity over the patient’s lifetime. Given this variability, regular patient evaluations are required to ensure that therapy is appropriate, being used correctly, aligns with individual patient needs, and potential side effects are minimized. A recently published guideline outlines an approach to monitoring attack frequency and severity in conjunction with regular visits with an HAE specialist [15].

Perhaps one of the most challenging areas in patient treatment is deciding when to start, switch, or stop LTP therapy. Although guidelines exist on which agents to consider when starting LTP, there are limited data comparing HRQoL, benefits, or risks with the use of LTP versus on-demand therapy only [304]. Given the clinical importance of these treatment decisions, in the absence of extensive evidence, the Conference Participants attempted to determine which variables should be considered when trying to decide when to start, switch, or stop LTP.

Recommendation 45 **The decision to start or stop long-term prophylaxis depends on multiple factors and should be made by the patient and an HAE specialist.**

**Level of Evidence: Consensus** (100% Agree)

**Strength of Recommendation: Strong** (100% Agree)

#### Clinical considerations

There was extensive discussion of the factors to consider when selecting on-demand or LTP treatment. Beyond general considerations (eg, availability of a therapy in the healthcare system, cost/reimbursement for the therapy, and access to the therapy despite potential barriers such as financial constraints or lack of insurance coverage), the factors that were viewed as important for both acute therapy and LTP included patient age and preference, route of administration, efficacy, and safety (eg, side effect profile). Conference Participants reported that ease of administration is strongly considered when selecting an acute therapy, while frequency and severity of attacks, comorbidities, contraindications, and family planning are strongly considered when selecting an LTP. The primary factors involved in decisions to start LTP include the frequency and severity of attacks, patient age and preference, access to emergency services/acute treatment, the efficacy and safety of LTP, its impact on HRQoL, and ease of self-administration.

Although the aim of LTP is to reduce the number and severity of attacks, it does not completely eliminate the risk of attacks. Patients must be aware that starting LTP does not guarantee the absence of attacks and there remains a risk of severe attacks, which may be fatal. All patients must have a plan for on-demand treatment of attacks and have at least two doses of acute treatments available even while on LTP therapy. Additionally, all patients must be monitored to ensure the efficacy of LTP and track any potential side effects [15, 305].

When patients are starting LTP it is important to emphasize that LTP is not necessarily a lifelong therapy and that treatment needs ongoing re-evaluation between the patient and HAE specialist. It may be helpful to try to objectively define patient expectations when starting LTP and review them regularly at follow-up visits. Part of the monitoring process should be to examine these goals and ensure they are being met.

The decision to switch LTP was also discussed. Key factors for considering a switch included lack of efficacy, side effects, route of administration, patient preference, changes in patient status (eg, pregnancy), and cost/reimbursement. Conference Participants highlighted the importance of balancing pharmacokinetics, patient preferences, safety, and practicality for smooth transition from one LTP to another. Tapering is recommended for androgen therapy and should be tailored to each patient’s preference [306]. However, tapering is not necessary for berotralstat, garadacimab, lanadelumab, or pdC1INH. Overlapping LTP therapies (based on medication half-life) ensures that the new therapy reaches a steady-state or sufficient efficacy before discontinuing the current treatment. Due to its rapid onset of action, an overlap may not be required when switching to garadacimab or pdC1INH, though this should be individualized for each patient [210]. A washout period for patients on androgen therapy is necessary to ensure safety and efficacy when transitioning to berotralstat. Rebound HAE attacks can occur after abruptly stopping androgens, and latent hepatotoxic effects of androgens can occur if berotralstat is started too soon after androgen discontinuation [307, 308]. Switching LTP therapies is a highly individualized decision [307]; considerations such as the route of administration, timing (including the need for loading doses), and convenience of the new therapy, as well as any potential anxiety about changes, are critical [306, 309, 310]. For patients who are pregnant or breastfeeding/lactating, it is essential to consider the safety profiles of overlapping medications and adjust therapy as needed (see section on **Long-term prophylaxis in pregnant, breastfeeding, or lactating patients with HAE**). The costs and affordability of overlapping treatments, should be factored in. Other considerations include ensuring a clear indication for switching and accounting for potential drug interactions that may affect efficacy or safety.

In accordance with **Recommendations 35 and 38**, LTP with androgens should be stopped immediately if a patient becomes pregnant or is breastfeeding/lactating. Additionally, several other factors should be considered when deciding to discontinue LTP. Patient preference plays a central role, particularly when patients have achieved complete symptom control or wish to stop LTP due to treatment fatigue or lifestyle changes. Side effects or reduced efficacy of the current therapy are often key considerations, as are the availability of newer treatments or initiation of clinical trials that may offer better management options. In many cases, concerns related to treatment access and cost/coverage are significant, particularly if the current therapy becomes unaffordable or unavailable once stopped. Additional factors include the patient’s ability to access emergency services/acute treatment if needed, potential drug interactions, and whether novel agents offer more effective alternatives. A diagnostic reconsideration that leads to a revised diagnosis may also prompt the decision to discontinue LTP. Ultimately, the decision to stop LTP should be guided by a comprehensive assessment of the patient’s clinical status, preferences, and access to care, ensuring that the transition is in the best interest of the patient. If the decision to reduce or stop LTP is made, the physician must ensure that the patient has ongoing access to the administration of appropriate on-demand therapy for attacks, as is consistent with **Recommendation 44**. All members of the patient’s comprehensive care team should be aware of the plan to reduce or stop LTP in case complications arise.

Most Conference Participants agreed that when stopping LTP, a gradual taper of androgen therapy is recommended (in the absence of serious side effects or pregnancy), whereas C1INH, lanadelumab, and berotralstat can either be stopped abruptly or administered at decreased frequency while monitoring the patient’s response. In all cases, the patient’s condition and access to on-demand therapy and appropriate follow-up care should guide the decision-making process.

The Conference Participants unanimously agreed that the decision to start or stop LTP should be made jointly by the patient and an HAE specialist. To enable an informed decision, the patient needs to be informed of the risks and benefits of all therapies, as discussed in the relevant sections above. Additionally, factors such as visit frequency, the provision of a written action plan, family and psychological support, multidisciplinary care (eg, communication with dentists, obstetricians), transition from pediatric to adult care, patient education, and laboratory monitoring must be considered for those on LTP.

### Quality of life

#### Background

The Constitution of the World Health Organization defines health as, “a state of complete physical, mental, and social well-being and not merely the absence of disease or infirmity” [311]. It follows that the measurement of health and the effects of healthcare must include not only changes in the frequency and severity of diseases, but also an estimation of well-being and disease-specific HRQoL. The impact of HAE on a person’s HRQoL can be considerable. An earlier survey conducted in the US in 2004 revealed that 85% of patients were afraid of sudden airway closure, 75% experienced intolerable pain, and 53% were concerned about transmitting HAE to their offspring [312]. HAE continues to impose a substantial burden on patients’ daily lives, particularly for those with frequent attacks or poor disease control, as demonstrated in a more recent US survey. [313]. An international survey conducted in 2021–2022 examined the relationship between attack-free periods and HRQoL. Patients with longer attack-free intervals reported less anxiety (including fear of attacks), fewer psychological issues (eg, stress, fear, anxiety, depression), fewer missed days from work or school, and fewer limitations in social and physical activities [314]. Psychological distress is commonly experienced by HAE patients, who report higher rates of anxiety and depression compared with the general population [315].

The Burden of Illness Study conducted in Europe (Denmark, Germany, Spain) was one of the first to comprehensively examine the impact of HAE on daily activities, providing valuable insights into how the condition affects patients’ lives both during and between attacks [316–318]. Patients reported substantial anxiety related to fear of future attacks, travelling, and transmitting HAE to their children [318]. More recently, a multinational patient survey by Mendivil et al. (2021) found that patients with HAE experienced a mean of 12.5 attacks over a 6-month period, with 21.9% of attacks lasting ≥ 3 days [319]. As a result, patients experienced substantial impairment at work and school. On average, patients missed 3 h of work each week (based on a 40-h work week), had a 25% impairment in their ability to perform tasks at work, and experienced a 24% reduction in work productivity due to their condition [319].

Productivity impairment has also been reported in multiple registry-based studies and real-world data, including one of the most comprehensive European registry-based studies that has captured HAE-related school and work absenteeism [316, 320–323]. Absenteeism due to HAE attacks ranged from 5.9 to 31.1% of patients’ total work or school time, depending on attack severity [321]. The average number of missed days from work or school per year ranged from 1.7 to 19.9 days [321]. Fifty-one percent (*n* = 84) of patients indicated that HAE had hindered their career or educational advancement [316].

European studies using generic HRQoL instruments, such as the European Quality of Life 5-Dimensions questionnaire or the 36-Item Short Form Survey, have shown that HAE attacks negatively impact HRQoL [324–326]. Clinical trials employing both these generic instruments and angioedema-specific tools, such as the AE-QoL and AECT, have demonstrated improvements in HRQoL and disease control with LTP [202, 204, 205, 207, 208, 223–225, 227, 228, 266, 268, 327]. The AECT, which has been validated in adults and widely recognized as the gold standard for assessing HAE control, is simple to use and effective [328]. However, it is important to note that treatment of HAE and reduction in symptoms may not always correlate with significant improvements in HRQoL.

Several studies have focused on developing and validating disease-specific HRQoL tools to assess disease burden and disease control in HAE. The HAE-QoL questionnaire specifically evaluates the impact of HAE due to *C1INH* deficiency on adult patients’ HRQoL. It consists of 25 items across seven dimensions, with the global score reflecting the sum of all items and higher scores indicating better outcomes [329, 330]. This tool requires recall over a 6-month period [331] and has been used in clinical trials of LTP [233]. Even with disease-specific tools such as the HAE-QoL, usage is still limited compared with more general tools such as the AE-QoL.

The HAE-Activity Score (HAE-AS) is a retrospective measure, with a 1–6 month recall period, which assesses disease activity in patients with HAE due to *C1INH* deficiency [331, 332]. Although the HAE-AS has been validated in adults [332], a minimal clinically important difference has not yet been established.

The HAE-C1INH-QoL is a validated, HAE-specific HRQoL tool developed specifically for US patients with HAE due to *C1INH* deficiency. Its development adhered to US Food and Drug Administration guidance on patient-reported outcomes, ensuring regulatory compliance and methodological rigour. Additionally, it incorporated input from 415 US patients, reflecting a patient-centred approach and enhancing its relevance to the US healthcare context. Comprising 31 items across five domains, this instrument has demonstrated both validity and reliability. It identified attack frequency and severity as key factors influencing HRQoL. Compared to the AE-QoL, the HAE-C1INH-QoL contains items more pertinent to the experiences and concerns of US patients with HAE [333].

Recommendation 46 **Healthcare providers should routinely assess disease control, disease activity, and quality of life in patients with HAE using validated instruments in order to optimize HAE management.**

**Level of Evidence: Unable to reach consensus**

**Strength of Recommendation: Strong** (90.7% Agree, 9.3% Disagree)

#### Clinical considerations

A level of evidence was not assigned to the recommendation on HRQoL assessment, as participants could not reach consensus on the strength of the evidence, even though over 80% agreed with its wording. Although validated HRQoL instruments are commonly used in clinical trials, it remains unclear whether assessing HRQoL outside of clinical trials leads to better patient outcomes. Additionally, there is limited guidance on how to use HRQoL tools in the clinic, raising concerns about the potential burden of administering these instruments to patients. The recommendation was included nonetheless, due to its clinical importance.

As treatment options for HAE continue to expand, management strategies must be personalized to each patient’s clinical needs and preferences, considering their attack frequency/severity and HRQoL. Despite the availability of treatments, many patients feel their care does not fully address HRQoL concerns, highlighting the importance of considering patient experiences in treatment plans [334, 335].

Integrating patient-reported outcome measures (PROMs) into electronic medical record systems could streamline their use, benefiting both physicians and patients. However, challenges arise due to limitations in healthcare provider time and variations in platforms used across healthcare systems. Disease control should be assessed at each visit, whereas HRQoL could be evaluated at treatment initiation and after a defined period. Allowing patients to complete forms at home or while waiting to see their specialist could further enhance efficiency.

Currently, there is a gap in tools for pediatric populations, with no validated PROMs for children with HAE aged < 12 years. Additionally, there are limited data on PROMs for pregnant or breastfeeding individuals with HAE, as well as for those with HAE-nC1INH. These gaps highlight the need for more comprehensive, age-appropriate, disease-specific PROMs to fully capture the impact of HAE and its treatments across diverse patient groups.

### Shared decision-making

#### Background

Shared decision-making is a collaborative process where physicians and patients work together to evaluate the best available evidence and make informed decisions [336]. This approach empowers patients to consider their options and select those that align with their preferences, while HAE specialists provide the necessary information to guide their choices. Active patient involvement ensures that decisions reflect their values and experiences, as patients are the foremost experts on how HAE impacts their lives.

Shared decision-making is a key element of effective healthcare and has been shown to improve patient outcomes, enhance adherence, increase patient satisfaction, and reduce healthcare costs [337–339]. Moreover, this approach fosters trust, improves understanding of treatment risks and benefits, and optimizes patient experience. Shared decision-making is a continuous process that involves multiple discussions over time, proven to be more effective than single encounters [340]. Recognizing its value, the Committee has included a recommendation for shared decision-making in HAE care.

Recommendation 47 **Shared decision-making should be used to create a partnership between the physician and patient focused on individualized care.**

**Level of Evidence: Consensus** (100% Agree)

**Strength of Recommendation: Strong** (100% Agree)

#### Clinical considerations

A study assessing shared decision-making in HAE management showed strong agreement between patients and their physicians regarding the effectiveness of the decision-making process [341]. Most of the patient–physician pairs surveyed confirmed that they discussed treatment options, weighed risks and benefits, and made decisions together. These findings suggest that when shared decision-making is routinely applied in HAE management, it fosters high levels of engagement and mutual agreement [341].

Effective communication is critical for establishing a successful shared decision-making model, particularly in rare diseases like HAE [340]. Patients, often experts in their own symptoms and the impact of these symptoms on their daily life, should play an active role in decision-making, with guidance from their physicians. Shared decision-making should be seen as an ongoing, dynamic process, which has been shown to improve outcomes when practised consistently [334, 340]. Physicians who manage patients with HAE are encouraged to consider the three-phase, shared decision-making model proposed by Banerji et al. (2021) [334]. This model outlines the discovery, discussion, and decision phases, offering a structured approach that ensures the patient’s goals remain central to the treatment selection process.

To be effective, shared decision-making in HAE care also requires pre-visit planning and a commitment from physicians to patient-centred care [342]. Empowering patients to reflect on and express their preferences significantly enhances their engagement. Research shows that shared decision-making increases the likelihood of patients proactively requesting treatment switches, as demonstrated in the context of switching between LTP medications [342, 343]. Using shared decision-making allows for a treatment change that aligns with the patients’ comfort and concerns [343].

The primary goals of treatment are to eliminate HAE attacks and improve HRQoL, and should be reflected in the shared decision-making process. By involving patients in treatment decisions, HAE specialists can ensure that the chosen therapies not only address disease activity and disease burden but also align with the individual’s HRQoL objectives.

Integrating shared decision-making into HAE care, with an emphasis on individualized treatment, is consistent with other treatment guidelines for HAE and underscores its importance in optimizing patient care [14, 15].

Recommendation 48 **Payers must consider the opinion of the HAE specialist when evaluating reimbursement for treatment of patients with HAE, including HAE nC1INH.**

**Level of Evidence: Consensus** (85.7% Agree, 4.8% Disagree, 9.5% Abstain)

**Strength of Recommendation: Strong** (90.9% Agree, 2.3% Disagree, 6.8% Abstain)

#### Clinical considerations

The rarity of a rare disease such as HAE limits the feasibility of conducting large randomized controlled trials, making it difficult to gather comprehensive evidence [344]. This is particularly applicable to patients with HAE-nC1INH variants. HAE specialists, informed by real-world clinical experience and published data, are uniquely positioned to assess each patient’s specific needs [14, 15]. Their expertise allows them to identify the most effective treatments for individual patients. Therefore, it is crucial for payers to involve HAE specialists in reimbursement decisions. Considering the severity and unpredictability of HAE attacks, withholding access to approved therapies that can substantially improve QoL and reduce the frequency of debilitating attacks would be ethically unjustifiable. Such denial could worsen patient outcomes and increase healthcare costs [345]. Involving HAE specialists in reimbursement decisions helps ensure patients receive optimal care, potentially improving health outcomes and promoting more efficient use of resources.

### Comprehensive care

#### Background

Comprehensive patient care is based on integration of the organization, delivery, and management of services related to diagnosis, treatment, care, rehabilitation and health promotion. Many rare disease groups have adopted the comprehensive care model, for example hemophilia care has been provided through this model for decades. There is evidence in other rare diseases that this model results in treatment adherence, better patient outcomes, and reduced costs of care [297, 298, 346]. HAE is similar to other rare blood disorders, including hemophilia, as it is a potentially life-threatening, chronic condition that requires a highly specialized, multidisciplinary team to manage. However, HAE has distinct clinical features that warrant a dedicated comprehensive care framework tailored to meet the specific needs of these patients. The recommendation to provide comprehensive care for patients with HAE is well established and has been included in previously published guidelines [31]. The specific elements of comprehensive care for HAE were published previously and are listed in Table 4. In addition, the ACARE program launched by global HAE patient groups specifies 32 requirements on topics such as infrastructure, management, research, education, and advocacy that must be fulfilled to demonstrate excellence in the management of angioedema [177]. However, it is worth noting that the ACARE model does not include access to social workers, specialized diagnostic testing, or a 24-h emergency helpline, all of which are important elements to consider.

**Table 4:** Requirements for comprehensive care in the management of hereditary angioedema patients [31]

Comprehensive care requirements
*Best clinical treatment outcomes, including:*
a. A comprehensive care team made up of nurse coordinator, clinician, social worker, data manager, pain management specialist, genetic counsellor, and administrative support
b. Access to specialized diagnostic testing
c. Access to home treatment
d. A networked Patient Information System to facilitate product recalls—collect data on therapy outcome measures and safety, and facilitate participation in clinical trials
e. Access to clinical advances as they become available
f. Access to 24-h support
g. Access to up-to-date standards of care, including standardized wallet cards
h. Tracking and intermittent audit of quality outcomes including beneficial and adverse outcomes through secure, comprehensive and networked data management
*Education of patients and staff regarding:*
a. Responsible self/family care (home care model) with home and self-infusion/administration instruction and support
b. Developments in the cause, diagnosis, treatment, outcomes, and prognosis of hereditary angioedema
c. Changes in the administrative management of the clinic
*An environment conducive to research, including:*
a. Access to and support for clinical trials of new treatments
b. Access to and support for translational research in diagnosis and prognosis
c. Access to and support for psychosocial research such as quality of life studies

Recommendation 49 **Comprehensive care for all patients with HAE should be provided to optimize treatment and outcomes.**

**Level of Evidence: Low** (100% Agree)

**Strength of Recommendation: Strong** (100% Agree)

#### Clinical considerations

Three studies were identified on comprehensive care interventions for HAE outcomes. One retrospective cohort review examined the impact of an electronic treatment plan on acute HAE management. After its implementation in emergency departments, the time to first medication decreased (from 1.3 h to 0.9 h), the diagnosis of airway swelling declined (from 29 to 4%), HAE diagnoses increased (from 10 to 30%), and the use of C1INH more than doubled (from 29 to 61%) [347]. A specific characteristic of this study was the availability of the electronic treatment plan across all emergency departments in the area, which is a feature not commonly seen in most regions. Systematic implementation of an HAE management program at a single centre in Brazil resulted in a significant improvement in clinical severity. The mean number of attacks per month decreased from 1.15 before the intervention to 0.23 after 14 months [348]. In addition, there was an increase in the proportion of patients experiencing zero attacks (< 5% at baseline to 40% at 14 months), as well as improvements in HRQoL and psychiatric comorbidities (eg, anxiety, depression, stress) [348]. A key factor in these outcomes was the optimization of HAE management through the initiation, adjustment, or modification of LTP doses in about half of the study population. This approach likely contributed to the observed improvements and could potentially be replicated at specialized treatment centres. A cross-sectional study conducted at an ACARE centre in Canada indicated that centralized care models for HAE overcome geographic barriers, helping patients in rural areas access quality care and achieve outcomes comparable to those of patients in urban areas [349].

Although the importance of the comprehensive care model in HAE was affirmed unanimously by Guideline Authors, and specific recommendations have existed with respect to its requirements, this care model is not available to all patients with HAE and any organization may adapt the model to its own context. The core principles of comprehensive care should be consistent and equally accessible within and between countries. While HAE can be costly, inadequate or inappropriate management may lead to even greater overall healthcare burdens. The Guideline Authors affirmed that ongoing monitoring of comprehensive care programs (eg, ACARE) is essential to measure their efficacy as well as their impact on patients’ outcomes such as disease control, morbidity and mortality, hospital admissions, HRQoL, and economic measures.

Recommendation 50 **All patients with HAE should be informed about HAE patient associations.**

**Level of Evidence: Consensus** (100% Agree)

**Strength of Recommendation: Strong** (100% Agree)

#### Clinical considerations

Patient organizations play a key role in supporting HAE patients and their caregivers. These organizations advocate for access to management and treatment for HAE patients, not only to control their disease, but also to support them in fulfilling their potential at home, school, work, and in their relationships. All patients should be encouraged to join their local and international patient organizations.

### Registries

#### Background

Patient registries are a proven method for collecting long-term real-world data about the epidemiology of a disease, the patients’ disease characteristics, treatment patterns, adverse effects of treatment, and the effectiveness of treatment on clinical and HRQoL outcomes [350]. As per the European Organisation for Rare Diseases, the National Organization for Rare Disorders, and the Canadian Organization for Rare Disorders, patient registries should be recognized as a global priority in the field of rare diseases. Key principles of these organizations’ joint declaration for rare disease registries include: (1) registries should encompass the widest geographic scope possible, to maximize the patient population in whom to conduct fundamental, clinical, and translational research; (2) registries should include data directly reported by patients as well as healthcare professionals, or registries should be linked with corresponding biobank data; (3) interoperability and harmonization between different registries should be consistently pursued; and (4) sustainability should be guaranteed [351]. The Canadian Hereditary Angioedema Registry is an international, prospective, multicentre, observational, open-ended angioedema registry that was initiated in 2023 and is recruiting participants [352].

Recommendation 51 **Physicians should actively participate and encourage patients to enroll in an HAE registry.**

**Level of Evidence: Consensus** (95% Agree, 5% Disagree)

**Strength of Recommendation: Strong** (100% Agree)

#### Clinical considerations

Patients enrolled in an HAE registry will have a traceable timeline of their treatment with biologics and blood products (not just at the batch level but at the individual treatment level) as well as a means to capture drug safety data. An HAE registry, especially a global one, will also provide a growing repository of data for research in the field with the aim to improve patient care [352]. The Guideline Authors recognized that not all treatment centres will have the necessary resources required to contribute to registry data collection. Provisions should be made to facilitate patient identification and integrate data from existing sources (eg, electronic medical records, laboratory data, health technologies), in order to reduce the burden of data collection on physicians and foster their involvement in HAE registries [353].

## Implementation and future directions

Following publication, CHAEN will support implementation and dissemination of the updated HAE guideline through several initiatives. The guideline will be posted on the CHAEN public website, with a French translation to increase accessibility within Canada. Patient-facing materials, including wallet cards, will be updated, and previously approved continuing medical education (CME) presentations will be revised and delivered as accredited CME. Executive summaries will be shared with relevant stakeholders, and a webinar will be hosted for CHAEN members to review key updates and practical considerations. Efforts will also be made to engage a broader healthcare audience, including family physicians, emergency department clinicians, and nursing staff. These activities aim to support knowledge translation, promote guideline adoption, and improve care for patients with HAE worldwide.

## Conclusions

This update to the 2019 Canadian Hereditary Angioedema Guideline is a collaborative effort involving Canadian and international HAE experts and patient groups, coordinated by CHAEN. Its goal is to optimize the management of patients with HAE worldwide by providing contemporary, evidence-based recommendations to healthcare providers who manage or are likely to encounter patients with HAE in their practice.

## Supplementary information

Below is the link to the electronic supplementary material.

Supplementary Material 1
Additional File 1. HAE RCT Evidence Tables. Randomized Controlled Trials. Includes data extraction, quality assessments, and study reference codes and citations for randomized controlled trials.

Supplementary Material 2
Additional File 2. HAE Lower Quality Comparison Study Evidence Tables. Lower Quality Comparison Studies. Includes data extraction, quality assessments, and study reference codes and citations for lower quality comparison studies.

## Data Availability

No datasets were generated or analysed during the current study.

## Appendices

### Appendix 1: Search strategy

#### Database: Ovid MEDLINE

Search conducted on April 13, 2024 and September 9, 2024 by KLR.

Results already identified in the November 2018 search to support the previous guideline were manually removed from results. The inclusion criteria are below.

#### Inclusion criteria

- English-language publications
- Human subjects
- At least five subjects in each comparison group
- Original results published in an indexed journal (ie, no data from abstracts/posters or pooled data from multiple studies were included)
- Population: Patients diagnosed with type I or type II hereditary angioedema or hereditary angioedema with normal *C1INH* of any age, including pregnant and pediatric populations

- Intervention—addressed at least one of:
  1. Acute treatment: C1INH, rhC1INH (ruconest), kallikrein inhibitors (ecallantide, sebetralstat), bradykinin receptor antagonists (icatibant), antifibrinolytic drugs (tranexamic acid), solvent/detergent-treated plasma (SDP), fresh frozen plasma (FFP), frozen plasma (FP), epinephrine, corticosteroids, H1 antagonists, H2 antagonists
  2. Long-term prophylaxis: C1INH (IV or subcutaneous), attenuated androgens (danazol), antifibrinolytics (tranexamic acid), rhC1INH (ruconest), synthetic steroids (tibolone), e-aminocaproic acid (EACA), oral kallikrein inhibitors (berotralstat, avoralstat), plasma kallikrein inhibitors (lanadelumab), activated factor XII monoclonal antibody (CSL-312)
  3. Short-term prophylaxis: C1INH (IV or subcutaneous), attenuated androgens (danazol), antifibrinolytics (tranexamic acid), anabolic steroids, fresh frozen plasma (FFP), frozen plasma (FP)
- Comparison:
  4. Any, including placebo, regular treatment, or no intervention/comparison with baseline

- Outcome:
  5. Frequency or severity of attacks, or symptom relief including at least one of:
  6. Time to symptom relief (onset or complete resolution)
  7. Mean symptom complex severity score (MSCS score)
  8. Treatment outcome score (TOS)
  9. Attack duration
  10. Time to treatment
  11. Rebound/relapse
  12. Number of attacks (eg, median per year or per month)
  13. Health-related quality of life
  14. Work productivity activity index
  15. Depression scores
  16. Anxiety scores

*Note*: Intermediate outcomes, such as laboratory measures (eg, mean serum concentrations of functional C1INH) were not included. Pooled data from multiple studies were not presented in the evidence tables.

#### PRISMA flow diagram

RCT, randomized controlled trial. Adapted from Page et al. (2021) [354].

### Appendix 2: Levels of evidence and strength of recommendation

#### Determining levels of evidence

Levels of evidence were adapted from GRADE [32–34]. GRADE is considered *outcome centric*, and traditionally recommends a single rating (ie, high, moderate, low, very low) for each outcome across the full body of evidence. As in the previous guideline update, a single rating was assigned to the known body of evidence supporting each recommendation. If no published evidence was identified in an area, but Guideline Authors determined that it was important to make a recommendation, this was labelled as *consensus* (see Appendix Table 5).

**Table 5:** Levels of evidence. Adapted from Balshem et al. (2011) [33]

Quality level	Definition
High	We are very confident that the true effect lies close to that of the estimate of the effect
Moderate	We are moderately confident in the effect estimate: the true effect is likely to be close to the estimate of the effect, but there is a possibility that it is substantially different
Low	Our confidence in the effect estimate is limited: the true effect may be substantially different from the estimate of the effect
Very low	We have very little confidence in the effect estimate: the true effect is likely to be substantially different from the estimate of effect
Consensus	There is no published evidence, but the area is important and warrants a recommendation

#### Parameters that impact certainty in the evidence

All nonrandomized, unblinded trials were considered to be low-level evidence [33].

All randomized controlled trials were initially considered to be high-level evidence, and were downgraded based on the parameters in Appendix Table 6 [33].

**Table 6:** Parameters that impact certainty in the evidence. Adapted from GRADE Working Group [356]

Parameters	Examples of limitations	Effect on level of evidence^a^
Limitations of design/risk of bias (Cochrane Risk of Bias tool [35])	Lack of allocation concealment Lack of adequate sequence generation Lack of blinding Incomplete data Selective reporting Other limitations, such as stopping early	Majority of items not satisfied or not reported = downgraded by one level
Inconsistency	Large variation in effect Poor heterogeneity of results	Downgraded by one level
Imprecision of results	Small sample size Wide confidence intervals around the estimate of the effect Study is underpowered	Noted but not downgraded
Publication bias	Bias introduced due to significant industry funding	Noted but not downgraded (majority of studies were industry funded)
Indirectness/ generalizability	Study population or setting differs significantly from population of interest	Not applicable

^a^If it was determined that the limitation was significant, the level of evidence was downgraded by two levels.

#### Determining strength of recommendation

Strength of recommendation was also adapted from GRADE (Appendix Table 7) [32–34]. Recommendations could be either *strong* or *weak*.

**Table 7:** Strength of recommendation. Adapted from Andrews et al. (2013) [32]

Factors that strengthen a recommendation	Commentary
1. Quality of evidence	The higher the quality of evidence, the higher the likelihood that a Strong recommendation is warranted
2. Balance between desirable and undesirable effects	The larger the difference between the desirable and undesirable effects, the higher the likelihood that a Strong recommendation is warranted
3. Values and preferences	The more values and preferences vary, or the greater the uncertainty in values and preferences, the higher the likelihood that a Weak recommendation is warranted
4. Costs (resource allocation)	The higher the costs of an intervention—that is, the greater the resources consumed—the lower the likelihood that a Strong recommendation is warranted

The following factors were considered when deciding on a strong or weak recommendation:

- Uncertainty in the estimates of likely benefit, and likely risk, inconvenience, and costs
- Importance of the outcome that treatment prevents
- Magnitude of treatment effect.
- Precision of estimate of treatment effect
- Risks associated with therapy
- Burdens of therapy
- Risk of target event
- Costs
- Varying values

Appendix Table 8 lists potential implications of a strong or weak recommendation on physicians, patients, and policy makers.

**Table 8:** Implications of a strong or weak recommendation

Stakeholders	Strong recommendation	Weak recommendation
Physicians	Most patients should receive the recommended course of action	Different choices will be appropriate for different patients, and you must help each patient to arrive at a management decision consistent with their values and preferences
Patients	Most people would want the recommended course of action and only a small proportion would not	Most would want the recommended course of action, but many would not
Policy makers	The recommendation can be adopted as a policy in most situations	Policy making will require substantial debate and the involvement of many stakeholders

## Abbreviations

ACARE: Angioedema Centers of Reference and Excellence
ACE: Angiotensin-converting enzyme
AECT: Angioedema Control Ttest
AE-QoL: Angioedema Quality of Life
*ANGPT1*: Angiopoietin 1 gene
AOHQ: Angio-oedème héréditaire du Québec
*CPN1*: Carboxypeptidase N gene
C1INH: Complement 1 esterase inhibitor
C4: Complement component 4
CHAEN: Canadian Hereditary Angioedema Network
*CPN1*: Carboxypeptidase N gene
*DAB2IP*: DAB2 interacting protein gene
DPP-IV: Dipeptidyl peptidase IV
EU: European Union
FP: Frozen plasma
*F12*: Coagulation factor XII gene
FXII: Coagulation factor XII
FXIIa: Activated factor XII
GRADE: Grading of Recommendations Assessment Development and Evaluation
HAE: Hereditary angioedema
HAE-ANGPT1: HAE due to causative variant in *ANGPT1*
HAE-AS: HAE-Activity Score
HAE-C1INH-Type1: HAE due to quantitative deficiency in *C1INH*
HAE-C1INH-Type2: HAE due to dysfunctional deficiency in *C1INH*
HAE-nC1INH: HAE with normal *C1INH*
HAE-FXII: HAE due to causative variant in *F12*
HAE-PLG: HAE due to causative variant in *PLG*
HRQoL: Health-related quality of life
*HS3ST6*: Heparan sulfate-glucosamine 3-O-sulfotransferase 6 gene
ICMJE: International Committee of Medical Journal Editors
IU: International units
IV: Intravenous
*KNG*: Kininogen 1 gene
LTP: Long-term prophylaxis
*MYOF*: Myoferlin gene
OLE: Open-label extension
pd: Plasma derived
*PLG*: Plasminogen gene
PROM: Patient-reported outcome measure
QoL: Quality of life
RCAOH: Réseau Canadien d’angioédème héréditaire
rh: Recombinant human
RoB2: Cochrane Risk of Bias 2 Tool
SC: Subcutaneous
SDP: Solvent/detergent-treated plasma
*SERPING1*: Serpin family G member 1 gene
STP: Short-term prophylaxis
US: United States
